# A workflow to reduce red blood cell autofluorescence for imaging IL-4/IL-4R interactions in the inflamed lung

**DOI:** 10.1016/j.bbrep.2026.102678

**Published:** 2026-06-22

**Authors:** Cyril Salama, Alexandre Cousin, Myriam Oger, Diane Riccobono, Anne-Laure Favier, Krisztina Nikovics

**Affiliations:** a Imaging Unit, Department of Platforms and Technology Research; French Armed Forces Biomedical Research Institute, Brétigny-sur-Orge, 91223, France; b Radiobiology Unit, Department of Radiation and Bioeffects, French Armed Forces Biomedical Research Institute, Brétigny-sur-Orge, 91223, France

**Keywords:** Autofluorescence, Protein-receptor interaction, Immunostaining, Red-blood cells, IL-4, IL-4R, Proximity ligation assay (PLA), Spectral separation

## Abstract

**Motivation:**

Autofluorescence is a common challenge in fluorescence microscopy of inflamed tissue, particularly where red blood cell (RBC) autofluorescence can mimic true fluorescence signals. This complicates image interpretation, reduces counting accuracy, and undermines reproducibility. Reliable methods are needed to distinguish true signals from autofluorescence, while preserving tissue architecture. In this study, we address this challenge by developing an optimized workflow that integrates antigen retrieval, selective fluorophore choice, and spectral separation.

**Summary:**

Autofluorescence hinders the detection of protein interactions in inflamed tissues by generating signals indistinguishable from true positives, leading to misinterpretation and errors. This study aims to establish and validate a reproducible workflow that enables discriminating between RBC autofluorescence and true proximity ligation assay (PLA) signals in paraffin-embedded inflamed lung tissue. Our approach combines antigen retrieval, selective fluorophore excitation, spectral unmixing, and post-processing to minimize autofluorescence while maintaining tissue integrity. We demonstrate the workflow's efficacy using the PLA, a fluorescence-based technique in which antibody-coupled DNA probes generate a detectable signal only when two proteins are located within nanometer-scale proximity. Using this approach, we detected PLA signals suggesting spatial proximity between interleukin-4 (IL-4) and its receptor (IL-4R) on RBCs within inflamed lung tissue, a context in which RBC autofluorescence is particularly problematic.

## Highlights

• Red blood cell autofluorescence produces false-positive signals in inflamed lung tissue.
• An optimized workflow effectively reduces autofluorescence and enhances signal specificity.
• The IL-4/IL-4R interaction is identified on red blood cells using this method.

## Graphical abstract

**Figure:**
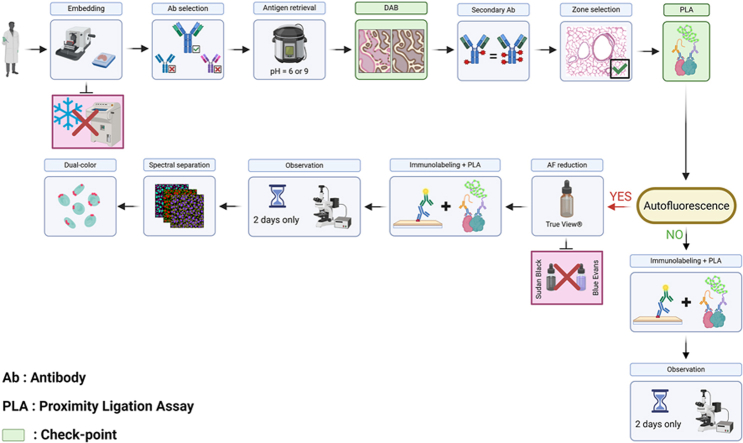

## 1 Introduction

Autofluorescence -the natural emission of light by cell components without labeling-is a pervasive challenge in fluorescence microscopy [[1], [2], [3], [4], [5], [6], [7]]. Various fluorophores, including NADH, FAD, collagen, elastin, lipofuscin, melanin, porphyrins, and lignin, contribute to autofluorescence [[8], [9], [10], [11]]. The autofluorescence problem is particularly complex in red blood cells (RBCs). RBCs display natural autofluorescence due to internal fluorophores present in their membranes and cytoplasms, such as heme-derived porphyrins, flavins, advanced glycation end products, and hemoglobin [12]. Hemoglobin, the oxygen-carrying protein in RBCs, contains heme as its active component. Heme consists of a porphyrin (protoporphyrin IX) and an iron ion (Fe^2+^). Porphyrins are ring-shaped molecules that can bind metals and fluoresce, thereby contributing to autofluorescence [13,14]. When RBCs are excited, typically in the blue-green wavelength range (approximately 350-500 nm), autofluorescence occurs, with emission in the green-red spectrum. RBCs can also be excited by red light (e.g., 647 nm) due to absorption by the protoporphyrin IX of hemoglobin, leading to emissions in the far-red range (around 660-720 nm) [15].

This phenomenon complicates image interpretation, leads to inaccurate quantification, and undermines the reproducibility of immunofluorescence-based assays, such as proximity ligation assays (PLA). While PLA is a powerful tool for visualizing protein-protein proximities (PPPs) *in situ* with high specificity and spatial resolution, it does not directly detect molecular binding. Instead, PLA signals indicate that the labeled proteins are in close proximity (typically within 40 nm), strongly suggesting that they may interact. This approach, therefore, enables the analysis of potential protein-protein interactions directly within tissues, including in contexts of high intrinsic autofluorescence. In inflamed lung tissue, red blood cells (RBCs) exhibit strong autofluorescence across a broad spectral range, which overlaps with the emission wavelengths of commonly used fluorophores and can compromise the detection of conventional fluorescence signals. In contrast, the proximity ligation assay (PLA) overcomes this limitation by generating an amplified fluorescent signal through rolling-circle amplification, thereby providing increased sensitivity and enabling reliable detection of protein-protein interactions even in highly autofluorescent environments. While individual strategies such as spectral unmixing, autofluorescence quenching, and optimized antigen retrieval for formalin-fixed paraffin-embedded samples have been proposed, a systematic and reproducible workflow integrating these approaches for PLA in highly autofluorescent tissues remains lacking. Optimized antigen retrieval is important because it enhances epitope accessibility and thereby improves the efficiency and specificity of antibody binding.

Interleukin-4 (IL-4) is a pleiotropic cytokine involved in Th2-mediated immune responses, allergic inflammation, and tissue repair [[16], [17], [18]]. It functions by binding to the IL-4 receptor (IL-4R), activating the downstream signaling pathways that regulate immune cell differentiation, antibody class switching, and macrophage polarization [[19], [20], [21], [22]]. While interactions between IL-4 and IL-4R have been well characterized in immune cells [19,20,23,24], their detection in red blood cells (RBCs) -traditionally regarded as lacking cytokine receptors-remains both controversial and technically demanding. Notably, emerging evidence suggests that RBCs can serve as reservoirs or carriers for cytokines, which may partly explain the reported detection of IL-4 and IL-4R in RBCs [25]. In this study, we leverage the detection of IL-4/IL-4R interactions as a proof-of-principle to validate our workflow, with the caveat that additional independent validation is necessary to confirm functional receptor expression on RBCs.

This study provides a methodological framework for overcoming autofluorescence in immunolabeling and PLA (Table 1), with potential applications in diverse autofluorescent tissues. By addressing a critical technical limitation, our workflow paves the way for more accurate and reproducible detection of protein interactions in complex biological samples.

**Table 1:** Alternative methods for detecting specific labeling by fluorescence or PLA against a complex background of autofluorescence.

Strategy	Technique/Tool	How It Works	Benefit for IF/PLA	Practical Notes
1. Amplify Specific Signal	- High-brightness fluorophores - Strong-affinity anti-cytokine antibodies - Optimized antigen retrieval	- Bright fluorophores emit a higher intensity of fluorescence - High-affinity antibodies bind more rapidly and with higher density - Gentle retrieval preserves epitopes	- Signal intensity versus autofluorescence → Clearer detection - Reduces need for extensive post-processing	- Balance fluorophore brightness against photostability - Lower retrieval pH can damage delicate cytokines - Titrate retrieval duration
2. Reduce Autofluorescence	- Sudan Black B - TrueVIEW Autofluorescence Quenching - Evans Blue - Paraffin embedding (preferred for cytokine retention) - Cryo-fixation (OCT) as a control	- Pigment-binding dyes or chemical quenchers mask or absorb the autofluorescence - Paraffin: fixation + embedding; may increase autofluorescence - Cryo: rapid freezing; preserves native state, lower autofluorescence	- Lower background intensity in the green -red spectra - Improves signal-to-noise for both fluorophores and PLA puncta - Paraffin: good morphology, higher background - Cryo: lower autofluorescence; partial loss of soluble proteins	- Effectiveness is variable – complete elimination is difficult - May slightly quench specific fluorophores; test controls are needed - Consider hybrid protocols (e.g., short destaining of paraffin sections) to reduce background
3. Distinguish Specific Fluorescence from Background	- Spectral unmixing - Dual-color immunofluorescence (image 1 –autofluorescence, image 2 – autofluorescence + specific fluorescence)	- Deconvolves overlapping emission spectra or mathematically removes background by image subtraction	- Allows accurate counting of cells displaying PLA labeling - Separates IL-4R signals from RBC autofluorescence	- Spectral unmixing is accurate but time-consuming - PLA subtraction is rapid and scalable

## 2 Materials and Methods

### 2.1 Materials and reagents

The following reagents were used: rabbit anti-human anti-IL-4 receptor polyclonal antibody (1 mg/mL, Abcam, cat. no. ab203398), mouse anti-human anti-IL-4 (IL-4-1) monoclonal antibody (1 mg/mL, Abcam, cat. no. ab239508), mouse anti-human anti-IL-4 (IL-4-2) monoclonal antibody (0.74 mg/mL, Thermo Fisher Scientific, cat. no. 66142-1-IG), mouse anti-human anti-IL-4 (IL-4-3) monoclonal antibody (200 μg/mL, Santa Cruz Biotechnology, cat. no. sc-12723), horse anti-rabbit IgG (H + L) antibody, DyLight™ 488 (1.5 mg/mL, Vector Laboratories, cat. no. DI-1088-1.5), donkey anti-rabbit IgG (H + L) highly cross-adsorbed secondary antibody, Alexa Fluor™ 488 (2 mg/mL, Thermo Fisher Scientific, cat. no. A-21206, RRID: AB_2535792), and donkey anti-mouse IgG (H + L) highly cross-adsorbed secondary antibody, Alexa Fluor™ 568 (2 mg/mL, Thermo Fisher Scientific, cat. no. A10037, RRID: AB_11180865), ImmPRESS HRP anti-rabbit IgG (peroxidase) polymer detection kit, made in goat (Vector Laboratories, cat. no. MP-7451, RRID: AB_2631198) and ImmPRESS HRP anti-mouse IgG (peroxidase) polymer detection kit, made in horse (Vector Laboratories, cat. no. MP-7402, RRID: AB_2336528), hemalum buffer (Merck, cat. no. 109249), phloxine B (Merck, cat. no. 15926), saffron (Sigma-Aldrich, cat. no. S8381), xylene (Sigma-Aldrich, cat. no. 247642), Sudan Black B (Sigma-Aldrich, cat. no. 199664), Evans Blue (Sigma-Aldrich, cat. no. E−2129), citrate-buffer L-recovery (pH 6.0, 10X, Aptum, cat. no. AP0533-500), e antigen retrieval solution (high pH 9.0, 10X, Invitrogen, cat. no. 00-4956-58), Triton X100 (Merck, cat. no. 112298), Tween 20 (Sigma-Aldrich, cat. no. P9416), PBS (Phosphate-Buffered Saline, without Ca and Mg, 10X, Eurobio, cat. no. GAUPBS0001), TBS-T (Tris-Buffered-Saline, 0.05% Tween 20, Vector Laboratories, cat. no. TWB945 M), and Antigenfix fixative (DiaPath, cat. no. P0014). Additionally, the following commercial assays and mounting media were employed: Fluoroshield mounting medium with DAPI (Abcam, cat. no. ab104139), Emerald antibody diluent (Sigma-Aldrich, cat. no. 936B-08), 3,3′-diaminobenzidine (DAB) substrate peroxidase (Eurobio, cat. no. K-4100), hydrogen peroxide solution (Sigma-Aldrich, cat. no. H1009), and an autofluorescence quenching kit (Eurobio, cat. no. SP-8400).

### 2.2 Animal model

All procedures complied with EU Directive 2010/63/EU and its French implementation (decree 2013-118), were approved by the French Army Animal Ethics Committee (N° 2011/22.1 and N° 506316/ARM/IGSSA/SP du 28 May 2019), and adhered to ARRIVE guidelines. Four 10.5-week-old Large-White/Landrace/Pietrain male pigs (≈27 kg) were used: a healthy negative control, two naturally infected pigs with lower respiratory-tract disease euthanized 5 days after symptom onset, and one pig exposed to an organophosphorus agent (OAE) with lungs harvested 6 h post-skin application. For our technical work, we specifically utilized the pig exposed to OAE. Anesthesia and euthanasia were achieved with an intravenous dose of 10 ml Dolethal (182.2 mg ml^−1^ pentobarbital, 10.4 mg ml^−1^ benzyl alcohol, 0.01 mg ml^−1^ cochineal red A); additionally, they received an intraperitoneal pentobarbital overdose. After euthanasia, lungs were excised, rinsed in sterile saline, and dissected into individual lobes. Portions were fixed in 10% neutral buffered formalin for histopathologic analysis, while adjacent sections were snap-frozen in liquid nitrogen for molecular analyses, ensuring standardized processing across all experimental groups.

### 2.3 Histological staining

All paraffin-embedded lung tissues used in this work were sectioned at 5 μm using a Leica HistoCore microtome; each tissue yielded 30 slides (2 sections per slide). Three independent replicates were performed for morphology, immunostaining, and PPP analyses, with representative images selected from each experiment. Sections were deparaffinized by consecutive 5-min washes in xylene (×2), 100% ethanol (×2), 95% ethanol (×2), and distilled water, then rehydrated with water. Hematoxylin-phloxine-saffron (HPS) staining was carried out by immersing the sections first in a 40-s hemalum buffer (0.2 g hemalum, 5 g Al_2_(SO_4_)_3_·xH_2_O in 100 mL H_2_O), followed by a 3-min water rinse, a 30-s phloxine dip (0.5 g phloxine in 100 mL H_2_O), a 1-min water rinse, and a 2-min 70% ethanol step; the latter was followed by 30 s in 95% ethanol and 1-min in 100% ethanol, repeated twice. Sections were then placed in a 10-min saffron buffer (6 g saffron in 200 mL absolute ethanol) and rinsed in absolute ethanol. This protocol yields blue-stained nuclei, pink cytoplasm, and orange connective tissue, enabling clear assessment of inflammatory lesions in all pig lung specimens.

### 2.4 Immunolabeling

Immunolabeling was performed on 5-μm paraffin-embedded lung sections obtained as serial sections adjacent to those used for HPS staining. Following deparaffinization and rehydration, antigen retrieval was carried out using a 15-min steam oven incubation in 10 mM citrate buffer (pH 6.0), followed by Tris-EDTA buffer (pH 9.0) to optimize antibody binding. Sections were permeabilized with 0.5% Triton X-100 in PBS for 15 min, rinsed three times, and blocked with Emerald Antibody Diluent for 1 h to minimize non-specific binding.

Primary antibodies (anti-IL-4, 1:500; anti-IL-4R, 1:200) were applied overnight at 4 °C in Emerald Antibody Diluent. After washing (3 × 10 min in PBS), sections were processed for either chromogenic or fluorescent detection.

For HRP-DAB staining, endogenous peroxidase activity was quenched with 10% H_2_O_2_ for 20 min. Sections were then incubated with species-specific secondary antibodies (1:1000) for 2 h at room temperature, followed by DAB substrate development and hematoxylin counterstaining.

For immunofluorescence, sections were incubated with Alexa Fluor 488- or DyLight 488-conjugated anti-rabbit secondary antibodies and Alexa Fluor 568-conjugated anti-mouse secondary antibodies (1:1000) for 2 h at room temperature. After washing, sections were mounted using DAPI-containing mounting medium.

Imaging was performed using a Leica Stellaris SP5 confocal microscope. Fluorophores were excited sequentially using laser lines at 405 nm (DAPI), 488 nm (Alexa Fluor 488 or DyLight 488), 568 nm (Alexa Fluor 568), and 647 nm (autofluorescence detection). Sequential acquisition was used to minimize spectral bleed-through. Emission was collected at 415-480 nm (DAPI), 500-550 nm (Alexa Fluor 488/DyLight 488), 570-620 nm (Alexa Fluor 568), and 660–720 nm (autofluorescence channel).

Within each experiment, identical acquisition parameters (laser power, detector gain, pinhole size, and scan settings) were maintained across samples to allow comparison. The pinhole settings were maintained at 1 Airy unit for all channels. Detector gain was adjusted initially to avoid signal saturation and then kept constant for all images within the same experimental set. Brightness and contrast adjustments were applied uniformly across images for visualization purposes only.

Spectral overlap between fluorophores and autofluorescence was addressed using LAS X spectral unmixing tools (Leica Microsystems). Lambda stacks (emission range: 485–650 nm) were acquired across the emission spectrum for each sample using 12 spectral channels sampled at 15 nm intervals. Reference emission spectra were obtained from regions enriched in specific labeling (IL-4R-positive regions) and from RBC autofluorescence-dominated regions. Linear unmixing was then performed on a pixel-by-pixel basis to separate overlapping signals.

### 2.5 Proximity ligation assay (PLA)

Proximity Ligation Assay (PLA) was performed to detect protein-protein proximity *in situ*, such as between cytokines and their receptors. While co-localization alone cannot prove interaction, PLA uses paired primary antibodies that bind the target proteins. Secondary “probe” antibodies, carrying single-stranded DNA strands (PLUS and MINUS), are then applied. When the two protein targets are within ∼40 nm, the attached DNA strands are brought into proximity. A hybridizing connector oligonucleotide ligates these strands into a closed circular DNA template. This template is then amplified by rolling-circle polymerase amplification. Fluorescently labeled probes are hybridized to the amplified product, yielding discrete fluorescent puncta that represent the interaction sites. Following antigen retrieval and permeabilization as described above, sections were processed using the NavienFlex Tissue Mouse/Rabbit kit (NT.MR.100 Red/Atto647 N) according to the manufacturer's instructions.

Briefly, sections were incubated with blocking solution (1× Block NT (37 °C) for 60 min in a humidified chamber, followed by overnight incubation (4 °C) with primary antibodies diluted in Diluent 1X NT. Negative controls were processed in parallel with the omission of primary antibodies. PLA probes (PLUS and MINUS) were applied for 60 min at 37 °C, followed by ligation (30 min at 37 °C) and rolling-circle amplification (90 min at 37 °C). Detection was performed using fluorescently labeled probes (containing Alexa Fluor 568).

After washing, sections were mounted in DAPI-containing medium and imaged using a Leica Stellaris SP5 confocal microscope. Sequential acquisition was performed using excitation at 405 nm (DAPI), 568 nm (PLA signal), and 647 nm (autofluorescence). Emission was collected using the same spectral ranges as described above.

To ensure consistency, imaging parameters were kept constant within each experimental set. Autofluorescence from RBCs was monitored in the 647 nm channel to aid interpretation of PLA signals.

PLA signal intensity was empirically observed to decrease over time. Slides were therefore imaged within 48 h of staining and stored at −20 °C to preserve signal integrity.

### 2.6 Reduction of autofluorescence

Three approaches were used to reduce tissue autofluorescence to antibody labeling.

(1) Sudan Black B: 300 mg dissolved in 100 mL of 70% ethanol, incubated for 12 h at 37 °C, cooled, filtered, and applied to tissue sections for 30 min. Sections were washed in 70% ethanol (3 × 5 min) followed by PBS (30 min).
(2) Evans Blue: 10 mg dissolved in 100 mL of 1 × PBS, and applied to sections for 30 min, followed by PBS washes (3 × 5 min).
(3) Commercial Autofluorescence Quenching Kit: Applied according to the manufacturer's instructions.

All treatments were performed before antigen retrieval. This timing provided improved reduction of autofluorescence compared to post-labeling treatment.

### 2.7 Homology sequence analysis

Homology sequence analysis was performed with the Blastp program (NCBI) using IL-4 pig (AAA31055.1) and human (CAP72493.1); IL-4R pig (AAP23302.1) and human (CAA36672.1) protein sequences.

### 2.8 Quantification and statistical analysis

Quantitative analysis of IL-4/IL-4R co-localization and protein-protein proximity (PPP) signals was performed across three independent experiments. For each condition, three sets of 1000 DAPI-positive cells were analyzed per anatomical zone (four zones total), resulting in three technical replicates per sample.

Cell classification was performed manually in a blinded manner using predefined criteria applied consistently across all datasets. A cell was considered IL-4-positive or IL-4R-positive when fluorescence intensity exceeded the background level established from negative-control sections processed without primary antibodies. Co-localization was defined as the presence of overlapping IL-4 and IL-4R fluorescence signals within the same DAPI-positive cell in merged confocal images acquired under identical settings. Cells exhibiting overlapping IL-4 and IL-4R signals were classified as co-localization-positive. For PPP analysis, only discrete punctate PLA signals localized to DAPI-positive cells and absent from the autofluorescence reference channel were scored as positive events.

The frequencies of IL-4-positive, IL-4R-positive, co-localization-positive, and PLA-positive cells were presented as percentages of the total DAPI-positive cell population. Results are presented as mean percentages ± standard error of the mean (SEM).

Statistical analysis was performed using one-way ANOVA followed by Tukey's post hoc test. A p-value <0.05 was considered statistically significant.

## 3 Results

### 3.1 Optimization of a workflow to overcome RBC autofluorescence in PLA

#### 3.1.1 Inflammation causes structural damage to the lung tissues

**Fig. 1:**
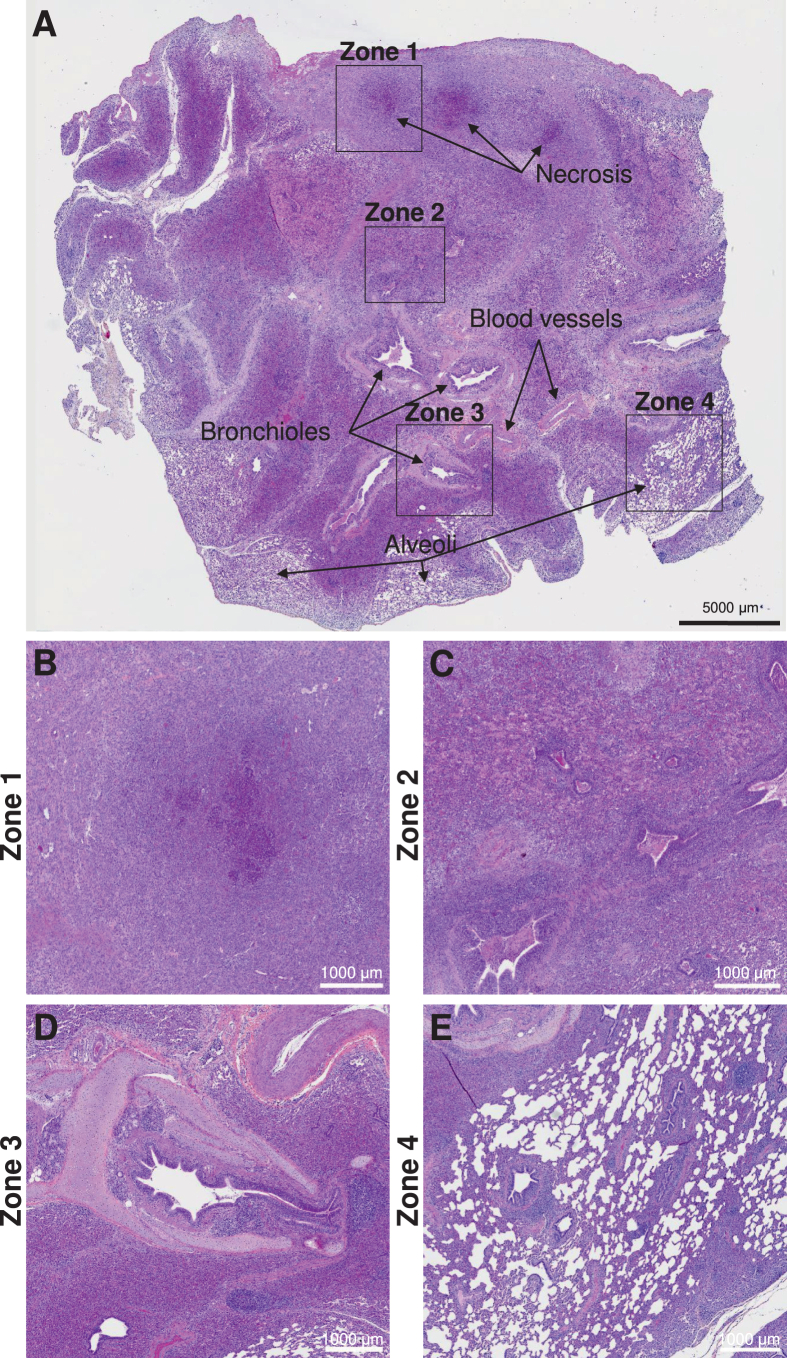
Histological characterization of the inflamed lung tissue. (A) Representative histological overview of inflamed lung tissue. (B) Zone 1: severe inflammation with extensive tissue necrosis. (C) Zone 2: inflamed tissue with macrophage infiltration and loss of alveolar structures. (D) Zone 3: inflammation localized in the bronchiolar region. (E) Zone 4: relatively preserved architecture with identifiable alveolar structures.

To establish a model for studying autofluorescence, we first performed histological analysis of inflamed porcine lung tissue. Samples were obtained from a porcine model, including two animals with naturally occurring lower respiratory tract infection and one animal exposed to an organophosphorus agent (OAE), a condition known to induce acute pulmonary inflammation. Initial optimization and technical analyses were performed using lung tissue from the OAE-exposed pig; subsequently, additional lung tissues were included for further validation. Four distinct zones were identified based on the extent of inflammation severity and structural damage: Zone 1 (necrotic area); Zone 2 (inflamed with macrophage infiltration); Zone 3 (bronchiolar inflammation); and Zone 4 (least damaged, with preserved alveolar structures (Fig. 1A–E).

#### 3.1.2 Amplification of a specific signal: antibody validation and antigen retrieval

To maximize signal detection, we optimized antibody selection, antigen retrieval, and fluorophore labeling.

##### 3.1.2.1 Antibody selection and validation

For studies of the expression of IL-4 and its receptor (IL-4R) in inflamed pig lung tissue, we first selected and validated appropriate antibodies by immunohistochemistry. Pig-specific reagents are scarce. We therefore screened human antibodies, making use of the high degree of amino-acid sequence identity between the porcine and human IL-4 (60%) and IL-4R (66%) proteins (Supplementary Fig. 1). Three commercially available anti-human IL-4 antibodies were screened for cross-reactivity with porcine IL-4 (Supplementary Fig. 2). IL-4-(1) demonstrated specific and localized staining pattern in inflamed lung tissue; whereas IL-4-(2) showed diffuse background staining comparable to control conditions (nonspecific staining), and IL-4-(3) showed no detectable signal (Supplementary Fig. 2A-C, F-H). Specificity was assessed based on signal localization, comparison with isotype and secondary-only controls, and correlation with inflamed regions. We therefore used IL-4-(1) as the antibody for IL-4 detection in all subsequent experiments. The anti-IL-4R antibody also displayed strong, specific staining (Supplementary Fig. 2D and I). Negative controls (omission of primary antibodies) confirmed the specificity of both antibodies (Supplementary Fig. 2E and J). We therefore considered the IL-4 and IL-4R antibodies to be validated for use in porcine lung tissue.

##### 3.1.2.2 Optimization of antigen retrieval

We compared low-pH citrate buffer (pH 6) and high-pH Tris-EDTA buffer (pH 9) for heat-induced epitope retrieval (HIER). Although both conditions produced IL-4 immunoreactivity, the Tris-EDTA buffer allowed faster visualization of the specific signal during DAB substrate development, requiring only 30 s compared with 45 s for the citrate buffer under identical staining conditions (Supplementary Fig. 2A and F). These results suggest improved epitope accessibility with high-pH retrieval, which was selected for subsequent experiments.

##### 3.1.2.3 Secondary antibody selection

We compared two fluorophores, DyLight488 and Alexa Fluor 488, conjugated to secondary antibodies under identical imaging conditions. No consistent differences in fluorescence signal intensity or background levels were observed across multiple representative fields and independent experiments (Supplementary Fig. 3). Given the comparable performance and the well-established photostability and signal consistency of Alexa Fluor 488, this fluorophore was selected for all subsequent immunofluorescence experiments.

#### 3.1.3 Reduction of autofluorescence: spectral unmixing and quenching strategies

To mitigate red blood cells (RBC)-associated autofluorescence and improve the reliability of fluorescence-based analyses, we evaluated three complementary approaches.

##### 3.1.3.1 Autofluorescence characterization and quenching

Fluorescence imaging of inflamed lung tissue revealed strong intrinsic autofluorescence associated with RBCs, interfering with specific antibody signals detection. To characterize this effect, we first analyzed tissue autofluorescence without antibody labeling using a DAPI-free mounting solution (Supplementary Fig. 4A–E). Under these conditions, RBC autofluorescence was detected in all imaging channels.

**Fig. 2:**
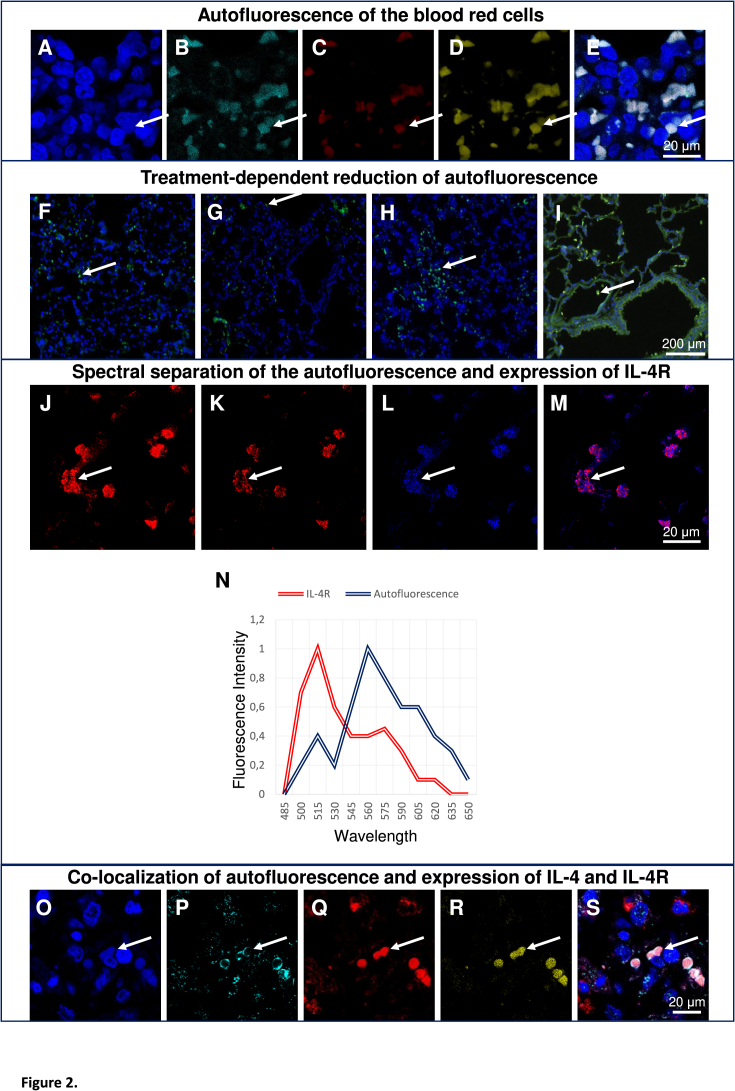
Characterization and reduction of tissue autofluorescence in inflamed lung tissue. (A-E) Representative fluorescence images of untreated inflamed lung tissue acquired at multiple excitation wavelengths (350, 488, 568, and 647 nm). Excitation at (A) 350 nm (blue), (B) 488 nm (cyan), (C) 568 nm (red), (D) 647 nm (yellow), and (E) a merged image of A-D. Intrinsic autofluorescence is observed throughout the tissue, with particularly strong signal associated with vascular structures containing red blood cells (RBCs). RBCs were identified based on combined morphological and spectral criteria. Morphologically, RBCs appear as small, round, intravascular, anucleated structures lacking DAPI-defined nuclear organization, in contrast to nucleated lung cells showing distinct nuclear staining patterns. In RBC-containing regions, the blue channel corresponding to DAPI excitation (350 nm) was not limited to nuclear staining but also included the intrinsic autofluorescence of RBCs. RBCs exhibit broad-spectrum autofluorescence across all excitation channels (350, 488, 568, and 647 nm), consistent with their known optical properties. (F–I) Evaluation of autofluorescence quenching strategies applied to lung tissue.: (F) Sudan Black B, (G) TrueVIEW autofluorescence quenching kit reduce general tissue background fluorescence, while (H) Evans Blue partially reduces autofluorescence but introduces additional far-red signal. (I) Autofluorescence of the untreated tissue. Autofluorescence is shown in green for visualization purposes. Blue staining corresponds primarily to nuclear DAPI labeling but also includes the intrinsic autofluorescence of red blood cells (RBCs). (J-N) Spectral separation of autofluorescence and antibody-associated signals (expression of IL-4R) using lambda stack acquisition and linear unmixing. Distinct emission profiles allow partial separation of RBC-associated autofluorescence from specific immunofluorescent signals: (J) Observation of the combined labeling of IL-4R and the autofluorescence of RBCs in inflamed lung tissue after excitation with 488 nm light (red). (K) Identification of IL-4R following spectral separation (red), and (L) observation of the autofluorescence of RBCs (blue). (M) A merged image combining K and L. (N) Microspectroscopic analysis of emission of Alexa Fluor 488 (blue line), autofluorescence (red line). (O–S) Dual-channel imaging strategy used to spatially resolve autofluorescence and antibody-derived signals across excitation channels. RBC-associated autofluorescence is consistently detectable across all channels, whereas specific antibody labeling shows distinct spatial patterns.: (O) Nuclear staining with DAPI together with RBC autofluorescence (blue color). (P) Expression of IL-4R (cyan color). (Q) Expression of IL-4 and autofluorescence of RBCs (red). (R) Autofluorescence of RBCs (yellow). (S) A merged image combining N-Q. White arrows indicate representative regions of erythrocyte-associated autofluorescence.

In a second experiment, the same analysis was performed using a mounting solution containing DAPI to visualize cell nuclei. Excitation at 350 nm enabled detection of both RBC autofluorescence and DAPI nuclear staining. In contrast, the other excitation channels showed only the intrinsic autofluorescence of RBCs (Fig. 2A–E).

Red blood cells were identified using a combination of morphological and spectral criteria. Morphologically, RBCs appeared as small, round cells within vascular lumens and lacked visible nuclei. In contrast, lung tissue cells exhibited characteristic DAPI-positive nuclear chromatin organization. Spectrally, RBCs displayed broad autofluorescence detectable at all tested excitation wavelengths (350, 488, 568, and 647 nm), consistent with the optical properties of hemoglobin.

Importantly, the signal detected in the DAPI channel (350 nm excitation) within RBC regions did not correspond to nuclear staining alone, but also reflected the intrinsic autofluorescence of RBCs. This interpretation was supported by the absence of nuclear morphology in these cells. Thus, RBC identification relied on: (i) the absence of nuclear structure despite DAPI excitation, (ii) their characteristic intravascular localization, and (iii) broad-spectrum autofluorescence across all imaging channels. This multiparametric approach enabled reliable discrimination of RBCs from nucleated tissue cells despite spectral overlap.

To reduce background fluorescence, several quenching strategies were evaluated without antibody labeling. Using 488 nm excitation, Sudan Black B (Fig. 2F) and the TrueVIEW Autofluorescence Quenching Kit (Fig. 2G) effectively reduced general tissue autofluorescence. Evans Blue (Fig. 2H) was also tested as a broad-spectrum absorber; however, due to its emission in the far-red range, it may interfere with detection in the 647 nm channel. Despite these treatments, erythrocyte-associated autofluorescence persisted and remained a major source of background signal (Fig. 2F–I). This persistence likely reflects the strong and broad emission characteristics of hemoglobin. These results indicate that while chemical quenching reduces general tissue autofluorescence, it is insufficient to fully eliminate RBC-associated signals.

##### 3.1.3.2 Spectral unmixing

To further improve signal discrimination, spectral unmixing was performed using lambda stack acquisition and linear unmixing algorithms (Fig. 2J–N). This approach relies on the principle that different fluorophores absorb and emit light at distinct wavelengths. By precisely controlling excitation and emission parameters, fluorescence signals can be separated based on their spectral profiles, enabling the discrimination of specific labeling from autofluorescence [26].

Emission spectra analysis revealed differences between antibody-associated fluorescence (peak ∼525 nm) and autofluorescence (broader emission centered around ∼550 nm) (Fig. 2N). These reference spectra were used for pixel-by-pixel linear unmixing to separate the two-component signal components.

While spectral unmixing improved signal discrimination, partial spectral overlap and variability in autofluorescence intensity prevented complete separation. Therefore, signal assignment was based on matching pixel emission profiles to the reference spectra of antibody-associated fluorescence and autofluorescence derived from control samples. This approach ensured that retained signals were consistent with the antibody-specific spectral signature, while minimizing misclassification of autofluorescence. Additionally, the method is relatively time-consuming and less suitable for high-throughput analyses.

##### 3.1.3.3 Dual-channel imaging

An alternative strategy approach was implemented using dual-channel imaging to distinguish autofluorescence from antibody-derived signals (Fig. 2O–S).

Autofluorescence associated with RBCs was monitored using 647 nm excitation, while IL-4R and IL-4 were detected using fluorophores excited at 488 nm and 568 nm, respectively (Fig. 2O–S). This approach enabled spatial identification of erythrocytes and facilitated comparison across channels.

RBC-associated autofluorescence was consistently detected in the far-red channel, although its apparent intensity varied across channels and imaging conditions, reflecting its broad emission spectrum. Using this approach, a signal consistent with IL-4R expression was observed at the surface of RBCs. However, no distinct IL-4-specific signal could be reliably assigned, as it was largely indistinguishable from RBC autofluorescence, as suggested in Fig. 2Q and S, preventing clear co-localization analysis.

#### 3.1.4 Application of the workflow: detection of IL-4/IL-4R interactions

Based on the above results described above, spectral unmixing and dual-channel imaging were selected for downstream co-localization and PLA analyses. Chemical quenching strategies (Sudan Black B, TrueVIEW kit, and Evans Blue) were excluded from the final workflow for the following reasons: (i) Ineffective RBC autofluorescence: despite effectively reducing general tissue autofluorescence, none of these treatments sufficiently attenuated erythrocyte-associated autofluorescence, which remained the dominant source of background signal; (ii) Spectral interference: some quenchers - notably Evans Blue - introduced additional spectral complications due to their own fluorescence emission; and (iii) Signal variability: Combining quenching with antibody labeling risked inconsistent signal intensity without a proportional gain in signal clarity. Spectral unmixing and dual-channel imaging, by contrast, operate on the acquired fluorescence signal rather than chemically altering the tissue, thereby preserving experimental consistency and enabling reliable post-acquisition signal discrimination.

To validate the workflow, we applied it to detect potential IL-4/IL-4R interactions using co-localization and PLA.

##### 3.1.4.1 Co-localization of IL-4 and IL-4R

**Fig. 3:**
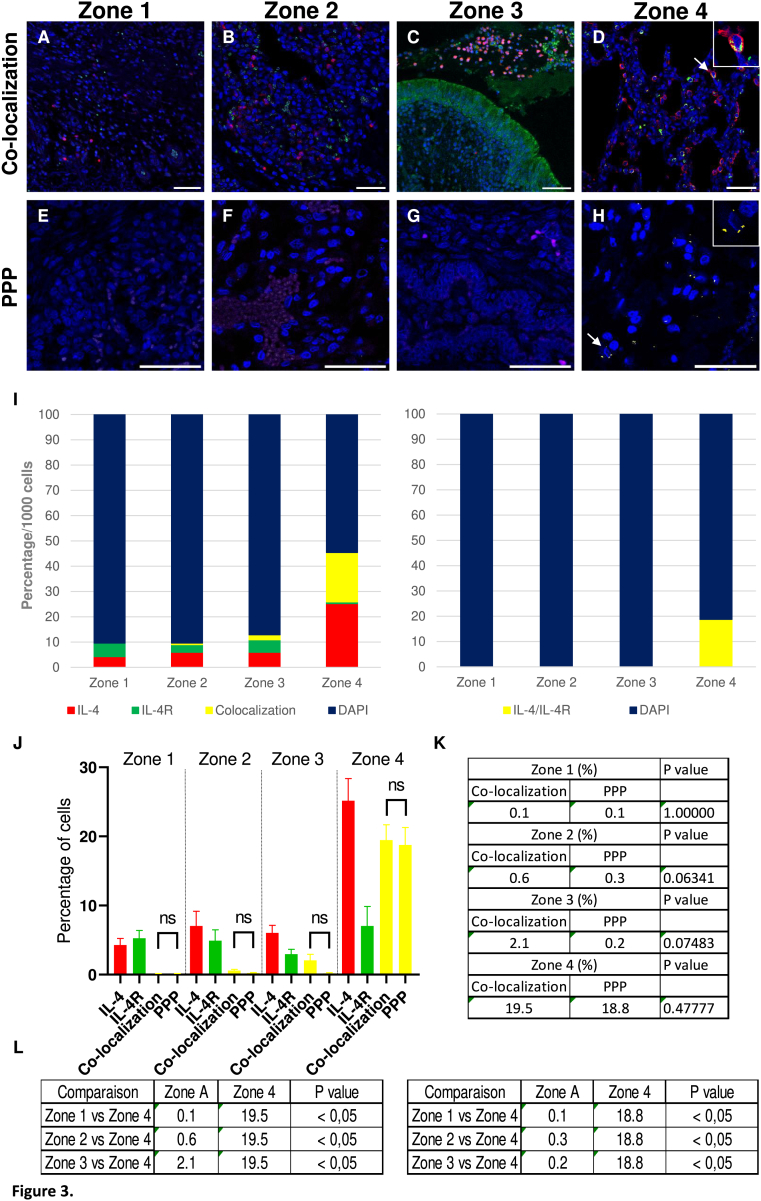
Co-localization and interaction between the IL-4 cytokine and the IL-4 receptor (IL-4R) in the inflamed lung regions. (A-D) Immunofluorescence detection of IL-4 and IL-4R across lung Zones 1-4. IL-4R was detected using Alexa Fluor 488 (green), and IL-4 using Alexa Fluor 568 (red). Co-localization appears as an overlapping signal (yellow). Nucleus (blue DAPI) excitation at 405 nm. (E-H) Proximity ligation assay indicating interactions between IL-4 and IL-4R. Signal consistent with proximity detected by PLA appears as punctate fluorescence (yellow). Nuclear staining was performed with DAPI (blue). Magenta signal in panels (E-H) corresponds to erythrocyte-associated autofluorescence excited at 647 nm. (A,E) Zone 1, (B,F) Zone 2, (C,G) Zone 3, and (D,H) Zone 4. Scale bar: 50 μm. White squares indicate higher-magnification insets of selected regions. (I-L) Quantitative analysis of IL-4 and IL-4R expression, co-localization, and PLA signals. A total of three independent sets of 1000 cells per zone were analyzed in a blinded manner across three independent experiments. All DAPI-positive cells within the defined regions of interest were included in the analysis, irrespective of IL-4 or IL-4R signal status. Cells were subsequently classified according to the presence or absence of IL-4 and IL-4R expression, their co-localization, or PLA signal. (I) Quantification of the percentage of cells exhibiting IL-4 or IL-4R expression, co-localization (left panel), or PLA signal (right panel) relative to the total DAPI-positive cell population in each zone. (J) Quantification of cells expressing only IL-4, only IL-4R, or displaying co-localization or PLA signal. (K) Comparison of co-localization and PLA frequencies within the same zone, showing no significant differences. (L) Comparison between Zone 4 and Zones 1–3. Data are presented as mean values from three independent experiments. Statistical analysis was performed using one-way ANOVA followed by Tukey's multiple comparison test. p < 0.05 indicates significant differences between Zone 4 and the other zones.

Co-localization of IL-4 and IL-4R was assessed across four anatomically distinct lung regions (Zones 1-4) (Fig. 3A–D). Imaging was performed using identical acquisition settings and sequential scanning to ensure comparability and minimize spectral bleed-through. A total of three independent experiments were analyzed, with 1000 cells per zone per experiment quantified manually in a blinded manner. Co-localization was quantified as the percentage of DAPI-positive cells exhibiting overlapping IL-4 and IL-4R fluorescence signals within the same cell. The frequencies of co-localization were calculated relative to the total DAPI-positive cell population in each zone. Co-localization was minimal in Zones 1 (0.1%), 2 (0.6%), and 3 (2.1%), but markedly increased in Zone 4 (19.5%). Negative controls (omission of primary antibodies) confirmed that background and autofluorescence signals did not produce significant false-positive co-localization (Supplementary Fig. 5A–D).

To further evaluate the specificity of IL-4R immunostaining, additional validation experiments were performed using lung tissues from two independent pigs with naturally occurring lower respiratory tract disease, as described in the Materials and Methods section. Both samples exhibited staining patterns comparable to those observed in the primary study, including prominent IL-4R-associated fluorescence in erythrocytes. Representative images from one animal are shown in Supplementary Fig. 6A and C, as similar findings were obtained in the second animal. As an additional specificity control, TNF and TNFR1 immunostaining were performed on lung tissues from pigs exposed to an organophosphorus compound. In contrast to IL-4R, neither TNF nor TNFR1 immunoreactivity was detected in erythrocytes, despite the use of comparable staining and imaging procedures. Representative images are presented in Supplementary Fig. 6B and D. Together, these findings support the conclusion that the erythrocyte-associated IL-4R signal is unlikely to result from nonspecific antibody binding or imaging artifacts.

##### 3.1.4.2 Confirmation of IL-4/IL-4R interactions

Proximity ligation assay (PLA) was used to assess IL-4/IL-4R interactions *in situ* (Fig. 3E–H). Distinct punctate signals were observed in all zones, with the highest frequency in Zone 4 (18.8%). The frequency of PLA signals was calculated as the proportion of DAPI-positive cells exhibiting at least one punctate PLA signal, relative to the total number of DAPI-positive cells in each zone. The spatial distribution of PLA signals was consistent with co-localization data, supporting the reliability of the workflow despite the presence of autofluorescence. No PLA signal was detected in negative controls (Supplementary Fig. 5E–H), confirming assay specificity.

**Fig. 4:**
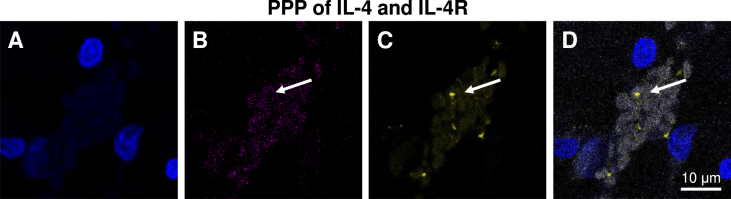
Detection of IL-4/IL-4R proximity signals in the context of RBC autofluorescence. (A) DAPI channel (blue). (B) Autofluorescence signal associated with RBCs (647 nm excitation, magenta). (C) PLA signal acquired using 568 nm excitation, displayed in yellow. (D) Merged image. Given the strong and broad emission spectrum of erythrocytes, a potential contribution of RBC autofluorescence to the 568 nm detection channel (C) cannot be fully excluded. Signal interpretation was therefore performed by directly comparing the PLA channel (C) with the dedicated autofluorescence channel (B): signals that co-localised spatially with the autofluorescence channel were treated with caution and excluded from quantification where possible. Only signals absent from the autofluorescence channel and consistent with the expected punctate PLA morphology were considered as true PLA events. White arrows indicate representative regions of erythrocyte-associated PLA signal.

Given the broad emission spectrum of RBC autofluorescence, a potential contribution to the 560 nm PLA detection channel (Fig. 4C) could not be fully excluded. To address this, PLA signal interpretation relied on direct comparison with the dedicated autofluorescence channel (Figs. 4B and 647 nm excitation). For quantification, a PLA punctum was considered valid only when no spatially overlapping signal was detected in the autofluorescence reference channel within the same cell area (i.e., absence of pixel-level co-localization in merged images at identical acquisition settings). Signals showing clear spatial overlap with autofluorescence were classified as autofluorescence-derived and excluded from quantification. Only punctate signals lacking autofluorescence overlap and displaying the characteristic morphology of PLA amplification products were scored as true positive events. This conservative approach may have led to a slight underestimation of true PLA events in erythrocyte-rich regions, but ensured that reported values reflect genuine proximity signals rather than background fluorescence.

##### 3.1.4.3 Quantification and statistical analysis

A total of three independent sets of 1000 cells per zone were manually analyzed in a blinded manner across three separate immunolabeled experiments. All nucleated DAPI-positive cells within the defined regions of interest were included in the analysis, irrespective of their IL-4 or IL-4R signal status. Erythrocytes were excluded from quantification despite exhibiting autofluorescence in the DAPI channel, based on their characteristic morphology (small size and distinct cellular shape), which allowed reliable discrimination from nucleated cells. Cells were subsequently classified based on the presence or absence of IL-4, IL-4R, and their co-localization or PLA signal. The frequencies of co-localization and PLA signals were quantified relative to the total DAPI-positive cell population for each zone (Fig. 3I). In a subsequent analysis, cells expressing only IL-4 or only IL-4R were quantified alongside cells exhibiting co-localization or protein proximity (PLA) signals (Fig. 3J). Data are presented as the percentage of positive cells, and statistical significance was determined using one-way ANOVA followed by Tukey's post hoc multiple comparison test. This analysis revealed no significant differences between co-localization and protein proximity (PLA) within the same zone (Fig. 3J and K). In contrast, statistically significant differences were observed between Zone 4 and each of the other zones (Zones 1–3) (p < 0.05) (Fig. 3L).

Although the interaction frequencies in Zones 1-3 were low, the consistent trend across biological replicates, together with the statistical significance observed for Zone 4, supports the robustness of these findings.

##### 3.1.4.4 Confirmation of IL-4/IL-4R interaction on red blood cell surfaces

To investigate the spatial proximity between IL-4 and its receptor (IL-4R) in erythrocytes, we employed a proximity ligation assay (PLA), which enables *in situ* detection of proteins located within close molecular proximities. Although PLA does not directly detect molecular binding, the detected signals indicate that the labeled proteins are located within distances compatible with a potential protein-protein interaction. Fig. 4A–D illustrates these results in detail. In Fig. 4A, DAPI labeling (blue) highlights the nuclei of cells present in the tissue as well as the autofluorescence of RBCs. In Fig. 4B, erythrocytes are visualized by their intrinsic autofluorescence, displayed in false-color magenta based on the corresponding emission channel (647 nm). Fig. 4C displays the PLA signal (yellow puncta; diffuse low signal in this channel is given by RBCs autofluorescence) corresponding to regions of close proximity between IL-4 and IL-4R. The merged image in Fig. 4D demonstrates spatial overlap between erythrocyte-associated autofluorescence and PLA-positive regions. Some PLA-positive puncta appeared associated with the erythrocyte periphery, consistent with membrane-associated localization, although the present imaging approach does not allow definitive assignment to the cell membrane. Together, these results demonstrate the applicability of PLA for visualizing protein proximities in highly autofluorescent biological environments.

## 4 Discussion

The visualization of cytokine-receptor complexes in lung tissue is significantly hampered by the intense autofluorescence of red blood cells (RBCs), which emit across a broad visible spectrum and overlap with the emission of most fluorophores used in immunofluorescence (IF) and proximity ligation assays [15,27,28]. This background reduces contrast and obscures true protein-protein interactions, posing a major technical challenge. This study presents a reproducible workflow to overcome RBC autofluorescence in PLA, enabling the detection of protein-protein proximity in autofluorescent tissues. By combining optimized antigen retrieval, selective fluorophore excitation, and spectral unmixing, we achieved a reduction in background fluorescence while preserving specific signal detection. This workflow addresses a critical technical limitation in fluorescence microscopy and provides a methodological framework for studying protein interactions in complex biological samples.

The tissue samples analyzed in this study originate from the same specimens previously used in our earlier work, where TNF-TNF receptor interactions were investigated using both Proximity Ligation Assay (PLA) and Surface Plasmon Resonance (SPR), yielding consistent results between the two independent approaches [29]. These experiments were performed during the same experimental series.

At the same time, we attempted to analyze IL-4/IL-4R proximity signals in the same tissue sections. However, strong autofluorescence associated with erythrocytes significantly interfered with the spectral analysis, limiting the reliability of IL-4/IL-4R signal interpretation. One of the motivations of the present technical report is precisely to document these methodological challenges encountered in erythrocyte-rich tissues when applying multiplex fluorescence and PLA approaches.

### 4.1 Technical strengths

The core strength of our approach lies in its multifaceted strategy, combining optimized antigen retrieval, selective fluorophore excitation, spectral unmixing, and autofluorescence subtraction. By using a Tris-EDTA buffer (pH 9.0) for antigen retrieval, we minimized damage to sensitive proteins while revealing masked epitopes. This gentler alkaline environment was critical for preserving cytokine and receptor epitopes, which are often compromised by harsher retrieval methods [[30], [31], [32]]. Higher pH buffers alter protein charge and hydrogen bonding, revealing previously masked epitopes [32]. By optimizing the retrieval conditions - specifically, by adjusting retrieval time and buffer choice - we were able to enhance both the sensitivity and specificity of cytokine immunostaining.

Autofluorescence does not significantly interfere if the emission intensity of the specific fluorophore is approximately two-to threefold higher than that of the autofluorescence. The exact ratio required can depend on the antibody; a twofold difference allows the signal to be distinguished, while a threefold difference makes the distinction clearer. In our experiments, detection of the IL-4 receptor was relatively straightforward because the signal of the fluorophore greatly exceeded autofluorescence (see Fig. 2P). However, IL-4 detection proved more problematic, as fluorophore emission and autofluorescence were of similar intensity (see Fig. 2Q). We then attempted to use antibodies with a strong affinity for the cytokine. We assessed the binding affinities of the antibodies by immunohistochemistry. We selected the IL-4-(1) antibody for its rapid signal detection (within 30 s) and consistent performance across experiments. This antibody was therefore employed consistently for all immunolabeling and PLA experiments in the study.

The cellular context of IL-4/IL-4R co-localization was not explicitly addressed in this manuscript. Based on our extended analyses, IL-4/IL-4R interactions appear to occur predominantly in erythrocyte-rich regions, where red blood cell autofluorescence posed significant technical challenges, as well as in areas enriched in immune cells, including macrophages and lymphocytes. Additional experiments on lung tissues from independently infected animals confirmed strong IL-4R expression in red blood cells, consistent with observations in the primary model. In parallel, TNFR1 expression was strongly detected in inflamed lung tissue but not in red blood cells, indicating a differential distribution of cytokine receptors. The presence of IL-4/IL-4R proximity signals in erythrocyte-rich regions is intriguing. Although this study primarily aimed to establish a robust workflow for detecting cytokine-receptor interactions in highly autofluorescent tissues, these findings may also have biological relevance. Erythrocytes have been described as dynamic reservoirs of cytokines and may contribute to their transport or sequestration within inflamed tissues [25]. In addition, the inflammatory microenvironment may favor transient cytokine accumulation in erythrocyte-rich areas [33]. However, the mechanistic basis of these interactions remains speculative and requires further investigation.

Due to technical limitations in combining proximity ligation assay with immunofluorescence, the precise cellular mediators of IL-4/IL-4R interactions could not be definitively identified. Moreover, their spatial distribution was not uniform: IL-4 expression and IL-4/IL-4R interactions were most prominent in Zone 4, where inflammation was less severe. This pattern is consistent with the anti-inflammatory role of IL-4 and suggests a potential contribution to localized modulation of inflammation and partial tissue regeneration.

Additionally, our use of spectral unmixing and PLA allowed us to distinguish true signals from background autofluorescence, providing a robust framework for studying protein interactions in complex biological samples. We initially used spectral unmixing, which effectively separated IL-4R signals from red blood cell (RBC) autofluorescence and revealed the presence of receptors on the surface of the RBCs. However, this method was time-consuming and impractical for routine use. We therefore explored alternative strategies, such as PLA, which was faster and simpler. This method involves capturing two types of images: one in which only RBC autofluorescence is recorded, and another displaying the overlap between the PLA signal and autofluorescence. By comparing these images, we were able to distinguish the true PLA signals from background autofluorescence.

Using PLA, we assessed the proximity of IL-4 and IL-4R in erythrocyte-rich regions of lung tissue. Although PLA signals were detected, overall co-localization was low, likely reflecting the combined effects of red blood cell autofluorescence and the spatial distribution of the molecules. The relatively low co-localization percentages observed in Zones 1–3 (0.1–2.1%) may in part reflect residual technical noise inherent to fluorescence-based detection in autofluorescent tissues, including incomplete spectral separation and signal variability between acquisitions. However, the substantially higher value observed in Zone 4 (19.5% co-localization; 18.8% PLA), combined with its statistical significance and reproducibility across three independent experiments, supports the interpretation that these signals represent a genuine biological phenomenon rather than a technical artefact. Nevertheless, a contribution of background signal to the measured values cannot be fully excluded, and quantitative results should therefore be interpreted with appropriate caution. Future studies incorporating non-autofluorescent model systems or single-molecule imaging approaches would help to further dissect technical noise from true biological signal. This analysis supports the utility of the proposed methodological workflow for differentiating PLA-derived signals from background autofluorescence in highly autofluorescent tissues, while acknowledging that some residual background contribution may remain.

The approach is reproducible across multiple samples and can be applied to other highly autofluorescent tissues. Future work could further optimize signal detection by reducing autofluorescence and improving spatial resolution.

### 4.2 Limitations

Paraffin-embedded tissues usually exhibit significantly higher background autofluorescence due to dehydration, clearing, and paraffin infiltration steps, which introduce chromophores and cross-linking artifacts that emit in the visible range. Cryo-fixed sections, such as those embedded in optimal cutting temperature (OCT) compound, showed lower autofluorescence and are generally more suitable for immunofluorescence applications [34]. However, cryo-fixation is associated with loss of low-molecular-weight secreted cytokines during processing [35,36]. For this reason, paraffin embedding was used in our workflow to ensure cytokine retention, while accounting for and mitigating the higher autofluorescence through spectral unmixing.

A potential limitation of our detection strategy is the inherent difficulty in visualizing secreted peptides and small proteins such as cytokines in tissue sections. Cytokines generally exist in two forms: one bound to the plasma membrane and the other released into the extracellular matrix. The duration of membrane binding varies significantly, directly affecting detection efficiency [9,37]. Cytokines that remain associated with the membrane for longer periods are easier to detect. By contrast, IL-4 is rapidly released into the extracellular environment, making its detection more difficult due to its rapid dissociation from tissues [38].

We tested multiple autofluorescence quenchers: i) Sudan Black B - a lipophilic dye that effectively quenches broad autofluorescence [15]; ii) the TrueVIEW Autofluorescence Quenching Kit, which has been reported to suppress endogenous fluorescence from non-lipofuscin sources, including aldehyde fixation, red blood cells, and structural elements, such as collagen and elastin [39], and iii) Evans Blue, which binds to hemoglobin and can reduce the autofluorescence [40]. However, as shown in Fig. 2F–I, none of these methods fully eliminated the autofluorescence, likely due to the broad emission spectrum of heme in RBCs. The initial quenching strategies were ineffective in sufficiently reducing the background signal and, in some cases, negatively impacted the quality of the tissue signal. As a result, these strategies were not included in the final experimental workflow. Instead, we concentrated on alternative approaches that proved to be more effective in our experimental setting. In the final experiments, we reduced autofluorescence using a combination of spectral unmixing, dual-channel analysis, and the proximity ligation assay (PLA). This approach provides high signal specificity through spatially restricted amplification. Together, these strategies enabled reliable detection of cytokine signals, even in the erythrocyte-rich regions with high intrinsic autofluorescence.

### 4.3 Applicability to other autofluorescent tissue

Our workflow is not limited to lung tissue. The principles of spectral unmixing, selective fluorophore excitation, and PLA can be adapted to other highly autofluorescent tissues, such as liver, spleen, or kidney, where RBCs or endogenous pigments pose similar challenges [9,[41], [42], [43], [44]]. The methodology's flexibility makes it a valuable tool for researchers across disciplines, including immunology, neuroscience, and materials science, where autofluorescence is a persistent issue [41,42]. A key advantage of our approach is its reproducibility. Standardizing antigen retrieval, imaging parameters, and post-acquisition analysis ensures reproducibility, while PLA offers a straightforward approach for detecting protein proximity signals in autofluorescent tissues, suggesting its potential applicability for similar experimental settings.

### 4.4 Future directions

This workflow has broad applicability beyond IL-4/IL-4R detection, particularly in tissues where autofluorescence poses a significant challenge, such as the liver, spleen, and brain. Future studies will focus on the following key areas.

#### 4.4.1 Methodological optimization for autofluorescent tissues

Workflow Adaptation: Optimize the protocol for other autofluorescent tissues by refining fluorophore selection and exploring complementary quenching strategies (e.g., combining spectral unmixing with targeted quenching agents).

Advanced Microscopy Techniques: Investigate the use of two-photon or light-sheet microscopy to further reduce autofluorescence and enhance spatial resolution, enabling deeper tissue imaging and more precise localization of protein interactions.

#### 4.4.2 Biological validation of IL-4/IL-4R interactions

Cell-Specific Immunostaining: Develop methods to confirm the presence of IL-4R on erythrocytes and other cell types, using multiplexed immunofluorescence combined with PLA.

Functional Assays: Assess whether IL-4/IL-4R interactions on RBCs influence cytokine signaling, immune modulation, or inflammatory responses, potentially through in vitro binding studies or ex vivo functional assays.

Independent Validation: It should be emphasized that the PLA and co-localization signals reported in this study indicate molecular proximity rather than confirmed functional receptor-ligand interactions. Independent validation using complementary approaches - such as flow cytometry to confirm IL-4R surface expression on erythrocytes, co-immunoprecipitation or pull-down assays to confirm physical association, or surface plasmon resonance to characterize binding kinetics - will be necessary to confirm the biological significance of the observed IL-4/IL-4R proximity signals. This is particularly important for signals associated with erythrocytes, given the unusual biology of these cells and the known technical challenges of working in erythrocyte-rich tissues.

#### 4.4.3 Comparative analysis of inflamed vs. non-inflamed tissues

Negative controls: The inclusion of non-inflamed lung tissue as a baseline control is essential to determine whether IL-4/IL-4R proximity signals are specifically associated with inflammatory conditions. Such controls will allow discrimination between constitutive and inflammation-induced interactions and are therefore critical for the correct interpretation of the observed signals. This point is acknowledged as an important consideration for future studies building on this work.

Quantitative Comparisons: Perform side-by-side analyses of inflamed and healthy tissues to evaluate differences in cytokine-receptor co-localization, expression levels, and spatial distribution.

#### 4.4.4 Expansion to other cytokine-receptor systems

Broader Applications: Apply this workflow to detect other cytokine-receptor interactions (e.g., IL-6/IL-6R, IFN-γ/IFN-γR) or protein complexes in inflamed tissues, expanding its utility in immunology and pathology research.

Disease Models: Explore the use of this methodology in preclinical models of inflammation, autoimmunity, or infection, where cytokine dynamics play a critical role.

By addressing these directions, we aim to improve workflow robustness and enhance understanding of cytokine–receptor interactions in both health and disease contexts.

## 5 Conclusion

This study addresses a critical technical limitation in fluorescence microscopy and provides a methodological framework for studying protein interactions in highly autofluorescent tissues. The proposed workflow does not eliminate autofluorescence but rather reduces and mitigates its impact, improving the ability to distinguish true biological signals from background fluorescence. Future work could further refine autofluorescence reduction strategies to enhance signal detection and improve the accuracy of PLA signal localization in highly autofluorescent, complex tissues. These improvements may expand the applicability of this workflow to a broader range of biological samples.

## Funding statements

This work was supported by the French Central Military Health Service and the General Delegation for Armament (DGA) (PDH2-NRBC-4-NR-4306).

## CRediT authorship contribution statement

**Cyril Salama:** Investigation. **Alexandre Cousin:** Investigation. **Myriam Oger:** Data curation, Validation. **Diane Riccobono:** Data curation, Funding acquisition, Methodology, Validation, Writing – review & editing. **Anne-Laure Favier:** Conceptualization, Data curation, Methodology, Supervision, Validation, Writing – original draft. **Krisztina Nikovics:** Conceptualization, Data curation, Methodology, Supervision, Validation, Writing – original draft.

## Declaration of competing interest

The authors declare the following financial interests/personal relationships which may be considered as potential competing interests: Diane Riccobono reports financial support and writing assistance were provided by French Central Military Health Service and the General Delegation for Armament (DGA). Krisztina Nikovics reports a relationship with French Central Military Health Service and the General Delegation for Armament (DGA) that includes: travel reimbursement. If there are other authors, they declare that they have no known competing financial interests or personal relationships that could have appeared to influence the work reported in this paper.

## Data Availability

Data will be made available on request.

## Appendix A Supplementary data

Multimedia component 1 Supplementary Figure 1. Amino-acid sequence similarity analysis for the pig and human (A) interleukin 4 (IL-4), and (B) interleukin 4 receptor (IL-4R) proteins.

Multimedia component 2 Supplementary Figure 2. Immunolabeling was performed with different antibodies and antigen retrieval buffers at pH 6 and pH 9, in regions of the inflamed lung. Panels (A-E) depict the 3,3′-diaminobenzidine (DAB) immunolabeling (in brown) of lung tissue after antigen retrieval at pH 6. Panels (F-J) show DAB immunolabeling with various antibodies (also in brown) of lung tissue after antigen retrieval at pH 9. The specific antibodies used were: (A, F) anti-IL-4-(1), (B, G) anti-IL-4-(2), (C, H) anti-IL-4-(3), (D, I) anti-IL-4 receptor (IL-4R). Panel (E,J) displays negative controls, to which no primary antibodies were added for immunolabeling. Nuclei were stained with hematoxylin, which appears blue/dark purple in the images. The scale bar represents 100 μm.

Multimedia component 3 Supplementary Figure 3. Immunolabeling of IL-4 receptor in inflamed lung regions. (A, C) Detection using DyLight™ 488-conjugated secondary antibody (green). (B, D) Detection using Alexa Fluor™ 488-conjugated secondary antibody (green). Nuclei were counterstained with DAPI (blue). Negative controls (absence of primary antibody) are shown in panels (C) and (D). White arrows indicate IL-4 receptor expression. Scale bar: 50 μm.

Multimedia component 4 Supplementary Figure 4. Visualization of intrinsic autofluorescence in the absence of antibody labeling. RBCs were identified based on morphology (small, round, anucleated cells within vascular lumens) and characteristic broad-spectrum autofluorescence. Signals were acquired following excitation at (A) 350 nm, (B) 488 nm, (C) 568 nm, and (D) 647 nm; (E) shows the merged image.

Multimedia component 5 Supplementary Figure 5. Negative controls for immunolabeling and PLA presented in Fig. 3. (A-D) Immunolabeling controls: (E-H) PLA controls (no primary antibodies). (A, E) Zone 1; (B, F) Zone 2; (C, G) Zone 3; (D, H) Zone 4. Signals in the absence of primary antibodies represent background and autofluorescence. IL-4R (Alexa Fluor 488, cyan), IL-4 (Alexa Fluor 568, red). Overlapping signals appear yellow. Autofluorescence (primarily erythrocytes) appears magenta. Nuclei (DAPI) appear blue. Scale bar: 50 μm.

Multimedia component 6 Supplementary Figure 6. In situ co-localization of IL-4/IL-4R and TNF/TNFR1 in inflamed lung regions. (A, C) Immunolabeling of IL-4 and IL-4R in naturally infected pigs with lower respiratory tract disease. (B, D) Immunolabeling of TNF and TNFR1 in Zone 4 of pigs exposed to an organophosphorus agent. IL-4R and TNFR1 were detected using Alexa Fluor™ 488 (green), while IL-4 and TNF were detected using Alexa Fluor™ 568 (red). Nuclei were counterstained with DAPI (blue). The white square indicates a higher-magnification inset of the region highlighted by the arrow in the main image. Scale bar: 50 μm.

## References

[1] Maceda A., Andrés-Hernández A.R., Terrazas T. (2024). Protocol to analyse the structural composition by fluorescence microscopy and different conventional and fluorescence staining methods. MethodsX.

[2] Maceda A., Terrazas T. (2022). Fluorescence microscopy methods for the analysis and characterization of lignin. Polymers.

[3] Jiang Y., Pu K. (2021). Molecular probes for autofluorescence-free optical imaging. Chem. Rev..

[4] Hwang W., McPartland T., Raymond T., Han M., Jeong S., Evans C.L. (2025). Quantitative investigation of photobleaching-based autofluorescence suppression in formalin-fixed paraffin-embedded human tissue. Biomed. Opt. Express.

[5] Hwang W., McPartland T., Jeong S., Evans C.L. (2025). A robust method for autofluorescence-free immunofluorescence using high-speed fluorescence lifetime imaging microscopy. Sci. Rep..

[6] Lee H.-E., Kim H.L., Cho A., Kim D.-G., Park M.-J., Oh Y., Park Y.S., Han R.T., Lee M.-Y., Riew T.-R. (2026). A practical workflow for fixation and autofluorescence reduction in correlative light and electron microscopy of postmortem human brain tissue. Tissue Cell.

[7] Schirripa Spagnolo C., Moscardini A., Amodeo R., Beltram F., Luin S. (2024). Optimized two‐color single‐molecule tracking of fast‐diffusing membrane receptors. Adv. Opt. Mater..

[8] Campbell J.M., Mahbub S.B., Anwer A.G., Habibalahi A., Gronthos S., Paton S., Grey S.T., Wu L.E., Gilchrist R.B., Goldys E.M. (2024). Multispectral imaging of collagen, NAD(P)H and flavin autofluorescence in mesenchymal stem cells undergoing trilineage differentiation. Cells.

[9] Liu Z., Meng J., Quinn K.P., Georgakoudi I. (2021). Tissue imaging and quantification relying on endogenous contrast. Adv. Exp. Med. Biol..

[10] König K., Teschke M., Sigusch B., Glockmann E., Eick S., Pfister W. (2000). Red light kills bacteria via photodynamic action. Cell. Mol. Biol. (Noisy-Le-Grand).

[11] Donaldson L. (2020). Autofluorescence in plants. Molecules.

[12] Stoya G., Klemm A., Baumann E., Vogelsang H., Ott U., Linss W., Stein G. (2002). Determination of autofluorescence of red blood cells (RbCs) in uremic patients as a marker of oxidative damage. Clin. Nephrol..

[13] Gell D.A. (2018). Structure and function of haemoglobins. Blood Cells Mol. Dis..

[14] Phillips J., Farrell C., Wang Y., Singal A.K., Anderson K., Balwani M., Bissell M., Bonkovsky H., Seay T., Paw B., Desnick R., Bloomer J. (2019). Strong correlation of ferrochelatase enzymatic activity with Mitoferrin-1 mRNA in lymphoblasts of patients with protoporphyria. Mol. Genet. Metabol..

[15] Whittington N.C., Wray S. (2017). Suppression of red blood cell autofluorescence for immunocytochemistry on fixed embryonic mouse tissue. Curr Protoc. Neurosci..

[16] Egholm C., Heeb L.E.M., Impellizzieri D., Boyman O. (2019). The regulatory effects of Interleukin-4 receptor signaling on neutrophils in type 2 immune responses. Front. Immunol..

[17] Pan D., Schellhardt L., Acevedo-Cintron J.A., Hunter D., Snyder-Warwick A.K., Mackinnon S.E., Wood M.D. (2022). IL-4 expressing cells are recruited to nerve after injury and promote regeneration. Exp. Neurol..

[18] Gieseck R.L., Wilson M.S., Wynn T.A. (2018). Type 2 immunity in tissue repair and fibrosis. Nat. Rev. Immunol..

[19] Junttila I.S. (2018). Tuning the cytokine responses: an update on interleukin (IL)-4 and IL-13 receptor complexes. Front. Immunol..

[20] Iwaszko M., Biały S., Bogunia-Kubik K. (2021). Significance of interleukin (IL)-4 and IL-13 in inflammatory arthritis. Cells.

[21] Song S.H., Kim J.E., Lee Y.J., Kwak M.H., Sung G.Y., Kwon S.H., Son H.J., Lee H.S., Jung Y.J., Hwang D.Y. (2014). Cellulose film regenerated from styela clava tunics have biodegradability, toxicity and biocompatibility in the skin of SD rats. J. Mater. Sci. Mater. Med..

[22] Jenkins S.J., Ruckerl D., Thomas G.D., Hewitson J.P., Duncan S., Brombacher F., Maizels R.M., Hume D.A., Allen J.E. (2013). IL-4 directly signals tissue-resident macrophages to proliferate beyond homeostatic levels controlled by CSF-1. J. Exp. Med..

[23] Ho I.-C., Miaw S.-C. (2016). Regulation of IL-4 expression in immunity and diseases. Adv. Exp. Med. Biol..

[24] Hoeksema M.A., Shen Z., Holtman I.R., Zheng A., Spann N.J., Cobo I., Gymrek M., Glass C.K. (2021). Mechanisms underlying divergent responses of genetically distinct macrophages to IL-4. Sci. Adv..

[25] Karsten E., Breen E., Herbert B.R. (2018). Red blood cells are dynamic reservoirs of cytokines. Sci. Rep..

[26] Rossetti B.J., Wilbert S.A., Mark Welch J.L., Borisy G.G., Nagy J.G. (2020). Semi-blind sparse affine spectral unmixing of autofluorescence-contaminated micrographs. Bioinformatics.

[27] Rey-Barroso L., Roldán M., Burgos-Fernández F.J., Gassiot S., Ruiz Llobet A., Isola I., Vilaseca M. (2020). Spectroscopic evaluation of red blood cells of thalassemia patients with confocal microscopy: a pilot study. Sensors (Basel).

[28] Garrido-Tamayo M.A., Rincón Santamaría A., Hoyos F.E., González Vega T., Laroze D. (2025). Autofluorescence of red blood cells infected with P. falciparum as a preliminary analysis of spectral sweeps to predict infection. Biosensors (Basel).

[29] Cousin A., Oger M., de Jenlis A., Lejart A., Barbier L., Riccobono D., Holy X., Favier A.-L., Nikovics K. (2025). CD163, a novel receptor for TNF, was revealed in situ by proximity ligation assay. Heliyon.

[30] Du J., Shi X., Zheng J., Zhou M., Cui X. (2005). [Antigen retrieval immunohistochemistry under the influence of pH value and time]. Beijing Da Xue Xue Bao Yi Xue Ban.

[31] Pileri S.A., Roncador G., Ceccarelli C., Piccioli M., Briskomatis A., Sabattini E., Ascani S., Santini D., Piccaluga P.P., Leone O., Damiani S., Ercolessi C., Sandri F., Pieri F., Leoncini L., Falini B. (1997). Antigen retrieval techniques in immunohistochemistry: comparison of different methods. J. Pathol..

[32] Krenacs L., Krenacs T., Stelkovics E., Raffeld M. (2010). Heat-induced antigen retrieval for immunohistochemical reactions in routinely processed paraffin sections. Methods Mol. Biol..

[33] Anderson H.L., Brodsky I.E., Mangalmurti N.S. (2018). The evolving erythrocyte: red blood cells as modulators of innate immunity. J. Immunol..

[34] Chen Y., Bi Y., Zhou C., Song J., Qi M., Gu Y., Tan Y., Qi J. (2025). Label-free histopathological diagnosis of frozen sections based on multi-excitation and broad-emission autofluorescence imaging. Biomed. Opt. Express.

[35] Soler P. (1995). [immunohistochemistry. What place should freeze drying of tissues be accorded?]. Ann. Pathol..

[36] Ramos-Vara J.A., Miller M.A. (2014). When tissue antigens and antibodies get along: revisiting the technical aspects of immunohistochemistry--the red, brown, and blue technique. Vet. Pathol..

[37] Liu G., Qi M., Hutchinson M.R., Yang G., Goldys E.M. (2016). Recent advances in cytokine detection by immunosensing. Biosens. Bioelectron..

[38] Spencer L.A., Melo R.C.N., Perez S.A.C., Bafford S.P., Dvorak A.M., Weller P.F. (2006). Cytokine receptor-mediated trafficking of preformed IL-4 in eosinophils identifies an innate immune mechanism of cytokine secretion. Proc. Natl. Acad. Sci. U. S. A..

[39] Sakr N., Glazova O., Shevkova L., Onyanov N., Kaziakhmedova S., Shilova A., Vorontsova M.V., Volchkov P. (2023). Characterizing and quenching autofluorescence in fixed mouse adrenal cortex tissue. Int. J. Mol. Sci..

[40] De la Lande I.S., Waterson J.G. (1968). Modification of autofluorescence in the formaldehyde-treated rabbit ear artery by Evans blue. J. Histochem. Cytochem..

[41] Croce A.C., Bottiroli G. (2014). Autofluorescence spectroscopy and imaging: a tool for biomedical research and diagnosis. Eur. J. Histochem..

[42] Monici M. (2005). Cell and tissue autofluorescence research and diagnostic applications. Biotechnol. Annu. Rev..

[43] Holz F.G. (2001). [Autofluorescence imaging of the macula]. Ophthalmologe.

[44] Schaefer P.M., Kalinina S., Rueck A., von Arnim C.A.F., von Einem B. (2019). NADH Autofluorescence-A marker on its way to boost bioenergetic research. Cytometry A.

