# Supplementary material for: A workflow to reduce red blood cell autofluorescence for imaging IL-4/IL-4R interactions in the inflamed lung
Source: Biochem Biophys Rep. 2026 Jun 22;47:102678. doi: 10.1016/j.bbrep.2026.102678 (PMC13316160; doi:10.1016/j.bbrep.2026.102678)
Supplement: Multimedia component 1 — Supplementary Figure 1. Amino-acid sequence similarity analysis for the pig and human (A) interleukin 4 (IL-4), and (B) interleukin 4 receptor (IL-4R) proteins. [file mmc1.pdf]

A

Pig: *Sus scrofa* interleukin 4 (IL-4)

```
>AAA31055.1 interleukin 4 [Sus scrofa]
MGLTSQLIPTLVCLLACTSNFVHGHKCDITLQEIIKTLNILTARKNSCMELPGDDVFAAPENTREKETFC
RASTVLRHIYRHHTCMKSLLSGLDRNLSSMANMTCSVHEAKKSTLKDFLERLKTIMKEKYSKC
```

Human: *Homo sapiens* interleukin 4 (IL-4)

```
>CAP72493.1 interleukin 4 [Homo sapiens]
MGLTSQLLPPLFFLLACAGNFVHGHKCDITLQEIIKTLNSLTEQKTLCTELTVTDIFAASKNTTEKETFC
RAATVLRQFYSHHEKDTRCLGATAQQFHRHKQLIRLLKRLDRNLWGLAGLNSCPVKEANQSTLENFLERL
KTIMREKYSKCSS
```

```
Pig    1    MGLTSQLIPTLVCLLACTSNFVHGHKCDITLQEIIKTLNILTARKNSCMELPGDDVFAAP  60
            MGLTSQL+P L  LLAC  NFVHGHKCDITLQEIIKTLN LT +K  C EL   D+FAA
Human  1    MGLTSQLLPPLFFLLACAGNFVHGHKCDITLQEIIKTLNSLTEQKTLCTELTVTDIFAAS  60

Pig    61   ENTREKETFCRASTVLRHIYRHHT----CMKS-------------LLSGLDRNLSSMANM  103
            +NT EKETFCRA+TVLR  Y HH     C+ +              L  LDRNL  +A +
Human  61   KNTTEKETFCRAATVLRQFYSHHEKDTRCLGATAQQFHRHKQLIRFLKRLDRNLWGLAGL  120

Pig    104  -TCSVHEAKKSTLKDFLERLKTIMKEKYSKC  133
             +C V EA +STL++FLERLKTIM+EKYSKC
Human  121  NSCPVKEANQSTLENFLERLKTIMREKYSKC  151
```

B

Pig: *Sus scrofa* interleukin 4 receptor(IL-4R)

```
>AAP23302.1 interleukin 4 receptor alpha [Sus scrofa]
MGWLCPGLTFSVSCLILVWAAGSGVTCVSPGGVRVLEWPICLSDYVSTSTCEWRMAGPVNCSAEFRLSYQ
LKFFNTENHTTCVPENRAGSVCVCHMLMESIVIVDTYQLDLWAGEQLLWNSSFKPSQNVKPLAPRNLMVH
ANISHTWLLTWSNPYPSESYLYSELTYLVNISNENDPTDFRIYNVTYLGPTLRFPANTLKSGAAYSARVK
AWAQRYNSTWSEWSPSVKWLNYYEEPLEQRLPLGVSISCVVILIICLSCYFGIIRIKKEWWDQIPNPAHS
PLVAIVIQDSQVSLWGKRSRGQEPAKCPRWKTCLTKLLPCFLEHGVDRDEDSSKAARNGPSQGPAKAAWR
PVEVSKTILWPESISVVRCVELFEAQVENEEEEEEEDKGSFCPSPENSGGSFQEGREGIAARLTESLFLD
LLGDESGAFSPQGMGQSCLLPPLENASAPMPWAEFPRVGSPEASSQGKEQPLNPEPSPQATPTQSLASLA
FPELPAVIADNPAYRSFSTFLSQSSDPGELDSDPELAEALEEVEPSLPAAPQPSEPPPTLQPEPETWEQI
LRQSVLQRRAAPAPASGPSSSGYREFVHAVEQGTQDRRAAGSGPCGEAGYKAFSSLLAGSASCPGTSGLE
PSSGESGYKPFQSLPPGCPETPVPTPLFTFGLDMEPPPSPQNPPFPGSSAECPGLEPAVKGEDGQKPPLA
LEQAADPLRDDLGSGIVYSALTCHLCGHLKQCHGQEDGGKVHVVASPCCSCCCEDGSPPMVTPLRAPDAP
SSGVPLEASLSPASLALLGVSREGKIPPCLQITPSNVQSSSQTPTAVAMLSPGPACMDTS
```

Human: *Homo sapiens* interleukin 4 receptor(IL-4R)

```
>CAA36672.1 interleukin 4 receptor [Homo sapiens]
MGWLCSGLLFPVSCLVLLQVASSGNMKVLQEPTCVSDYMSISTCEWKMNGPTNCSTELRLLYQLVFLLSE
AHTCIPENNGGAGCVCHLLMDDVVSADNYTLDLWAGQQLLWKGSFKPSEHVKPRAPGNLTVHTNVSDTLL
LTWSNPYPPDNYLYNHLTYAVNIWSENDPADFRIYNVTYLEPSLRIAASTLKSGISYRARVRAWAQCYNT
TWSEWSPSTKWHNSYREPFEQHLLLGVSVSCIVILAVCLLCYVSITKIKKEWWDQIPNPARSRLVAIIIQ
DAQGSQWEKRSRGQEPAKCPHWKNCLTKLLPCFLEHNMKRDEDPHKAAKEMPFQGSGKSAWCPVEISKTV
LWPESISVVRCVELFEAPVECEEEEEVEEEKGSFCASPESSRDDFQEGREGIVARLTESLFLDLLGEENG
GFCQQDMGESCLLPPSGSTSAHMPWDEFPSAGPKEAPPWGKEQPLHLEPSPPASPTQSPDNLTCTETPLV
IAGNPAYRSFSNSLSQSPCPRELGPDPLLARHLEEVEPEMPCVPQLSEPTTVPQPEPETWEQILRRNVLQ
HGAAAAPVSAPTSGYQEFVHAVEQGGTQASAVVGLGPPGEAGYKAFSSLLASSAVSPEKCGFGASSGEEG
YKPFQDLIPGCPGDPAPVPVPLFTFGLDREPPRSPQSSHLPSSSPEHLGLEPGEKVEDMPKPPLPQEQAT
DPLVDSLGSGIVYSALTCHLCGHLKQCHGQEDGGQTPVMASPCCGCCCGDRSSPPTTPLRAPDPSPGGVP
LEASLCPASLAPSGISEKSKSSSSFHPAPGNAQSSSQTPKIVNFVSVGPTYMRVS
```

```
Pig    1    MGWLCPGLTFSVSCLILVWAAGSGVTCVSPGGVRVLEWPICLSDYVSTSTCEWRMAGPVN  60
            MGWLC GL F VSCL+L+  A SG        ++VL+ P C+SDY+S STCEW+M GP N
```

```
Human  1    MGWLCSGLLFPVSCLVLLQVASSG-------NMKVLQEPTCVSDYMSISTCEWKMNGPTN  53

Pig    61   CSAEFRLSYQLKFFNTENHTTCVPENRAGSVCVCHMLMESIVIVDTYQLDLWAGEQLLWN  120
            CS E RL YQL F  +E HT C+PEN  G+ CVCH+LM+ +V  D Y LDLWAG+QLLW
Human  54   CSTELRLLYQLVFLLSEAHT-CIPENNGGAGCVCHLLMDDVVSADNYTLDLWAGQQLLWK  112

Pig    121  SSFKPSQNVKPLAPRNLMVHANISHTWLLTWSNPYPSESYLYSELTYLVNISNENDPTDF  180
             SFKPS++VKP AP NL VH N+S T LLTWSNPYP ++YLY+ LTY VNI +ENDP DF
Human  113  GSFKPSEHVKPRAPGNLTVHTNVSDTLLLTWSNPYPPDNYLYNHLTYAVNIWSENDPADF  172

Pig    181  RIYNVTYLGPTLRFPANTLKSGAAYSARVKAWAQRYNSTWSEWSPSVKWLNYYEEPLEQR  240
            RIYNVTYL P+LR  A+TLKSG +Y ARV+AWAQ YN+TWSEWSPS KW N Y EP EQ
Human  173  RIYNVTYLEPSLRIAASTLKSGISYRARVRAWAQCYNTTWSEWSPSTKWHNSYREPFEQH  232

Pig    241  LPLGVSISCVVILIICLSCYFGIIRIKKEWWDQIPNPAHSPLVAIVIQDSQVSLWGKRSR  300
            L LGVS+SC+VIL +CL CY  I +IKKEWWDQIPNPA S LVAI+IQD+Q S W KRSR
Human  233  LLLGVSVSCIVILAVCLLCYVSITKIKKEWWDQIPNPARSRLVAIIIQDAQGSQWEKRSR  292

Pig    301  GQEPAKCPRWKTCLTKLLPCFLEHGVDRDEDSSKAARNGPSQGPAKAAWRPVEVSKTILW  360
            GQEPAKCP WK CLTKLLPCFLEH + RDED  KAA+  P QG  K+AW PVE+SKT+LW
Human  293  GQEPAKCPHWKNCLTKLLPCFLEHNMKRDEDPHKAAKEMPFQGSGKSAWCPVEISKTVLW  352

Pig    361  PESISVVRCVELFEA-QVENEEEEEEEDKGSFCPSPENSGGSFQEGREGIAARLTESLFL  419
            PESISVVRCVELFEA     EEEE EE+KGSFC SPE+S   FQEGREGI ARLTESLFL
Human  353  PESISVVRCVELFEAPVECEEEEEVEEEKGSFCASPESSRDDFQEGREGIVARLTESLFL  412

Pig    420  DLLGDESGAFSPQGMGQSCLLPPLENASAPMPWAEFPRVGSPEASSQGKEQPLNPEPSPQ  479
            DLLG+E+G F  Q MG+SCLLPP  + SA MPW EFP  G  EA   GKEQPL+ EPSP
Human  413  DLLGEENGGFCQQDMGESCLLPPSGSTSAHMPWDEFPSAGPKEAPPWGKEQPLHLEPSPP  472

Pig    480  ATPTQSLASLAFPELPAVIADNPAYRSFSTFLSQSSDPGELDSDPELAEALEEVEPSLPA  539
            A+PTQS  +L   E P VIA NPAYRSFS  LSQS  P EL  DP LA  LEEVEP +P
Human  473  ASPTQSPDNLTCTETPLVIAGNPAYRSFSNSLSQSPCPRELGPDPLLARHLEEVEPEMPC  532

Pig    540  APQPSEPPPTLQPEPETWEQILRQSVLQRRAAPAPASGPSSSGYREFVHAVEQ-GTQDRR  598
             PQ SEP    QPEPETWEQILR++VLQ  AA AP S P +SGY+EFVHAVEQ GTQ
Human  533  VPQLSEPTTVPQPEPETWEQILRRNVLQHGAAAAPVSAP-TSGYQEFVHAVEQGGTQASA  591

Pig    599  AAGSGPCGEAGYKAFSSLLAGSASCPGTSGLEPSSGESGYKPFQSLPPGCPE--TPVPTP  656
              G GP GEAGYKAFSSLLA SA  P   G   SSGE GYKPFQ L PGCP    PVP P
Human  592  VVGLGPPGEAGYKAFSSLLASSAVSPEKCGFGASSGEEGYKPFQDLIPGCPGDPAPVPVP  651

Pig    657  LFTFGLDMEPPPSPQNPPFPGSSAECPGLEPAVKGEDGQKPPLALEQAADPLRDDLGSGI  716
            LFTFGLD EPP SPQ+   P SS E  GLEP  K ED  KPPL  EQA DPL D LGSGI
Human  652  LFTFGLDREPPRSPQSSHLPSSSPEHLGLEPGEKVEDMPKPPLPQEQATDPLVDSLGSGI  711

Pig    717  VYSALTCHLCGHLKQCHGQEDGGKVHVVASPCCSCCCEDGSPPMVTPLRAPDAPSSGVPL  776
            VYSALTCHLCGHLKQCHGQEDGG+  V+ASPCC CCC D S P  TPLRAPD    GVPL
Human  712  VYSALTCHLCGHLKQCHGQEDGGQTPVMASPCCGCCCGDRSSPPTTPLRAPDPSPGGVPL  771

Pig    777  EASLSPASLALLGVSREGKIPPCLQITPSNVQSSSQTPTAVAMLSPGPACMDTS  830
            EASL PASLA  G+S + K        P N QSSSQTP  V  +S GP  M  S
Human  772  EASLCPASLAPSGISEKSKSSSSFHPAPGNAQSSSQTPKIVNFVSVGPTYMRVS  825
```

Figure S1.
