# Supplementary figures and images for: A workflow to reduce red blood cell autofluorescence for imaging IL-4/IL-4R interactions in the inflamed lung
Source: Biochem Biophys Rep. 2026 Jun 22;47:102678. doi: 10.1016/j.bbrep.2026.102678 (PMC13316160; doi:10.1016/j.bbrep.2026.102678)

Citrate buffer (pH:6.0)

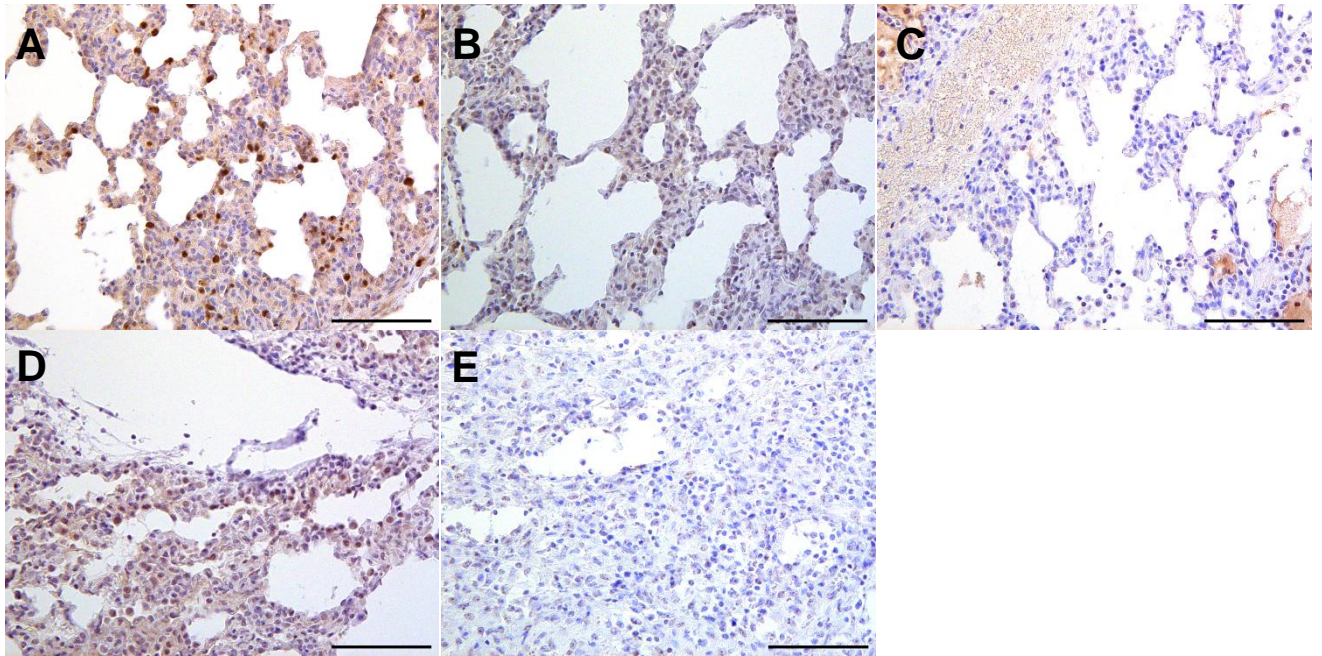

Tris-EDTA buffer (pH:9.0)

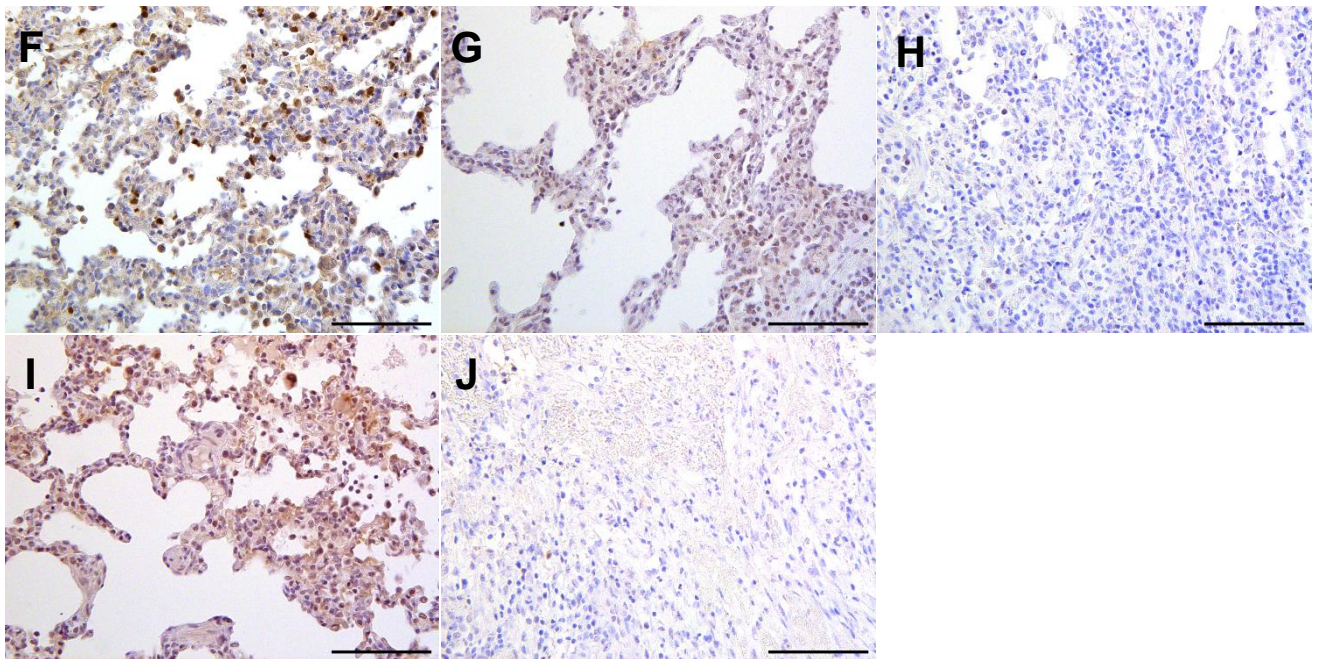

Figure S2.

Supplement: Multimedia component 2 — Supplementary Figure 2. Immunolabeling was performed with different antibodies and antigen retrieval buffers at pH 6 and pH 9, in regions of the inflamed lung. Panels (A-E) depict the 3,3′-diaminobenzidine (DAB) immunolabeling (in brown) of lung tissue after antigen retrieval at pH 6. Panels (F-J) show DAB immunolabeling with various antibodies (also in brown) of lung tissue after antigen retrieval at pH 9. The specific antibodies used were: (A, F) anti-IL-4-(1), (B, G) anti-IL-4-(2), (C, H) anti-IL-4-(3), (D, I) anti-IL-4 receptor (IL-4R). Panel (E,J) displays negative controls, to which no primary antibodies were added for immunolabeling. Nuclei were stained with hematoxylin, which appears blue/dark purple in the images. The scale bar represents 100 μm. [file mmc2.pdf]

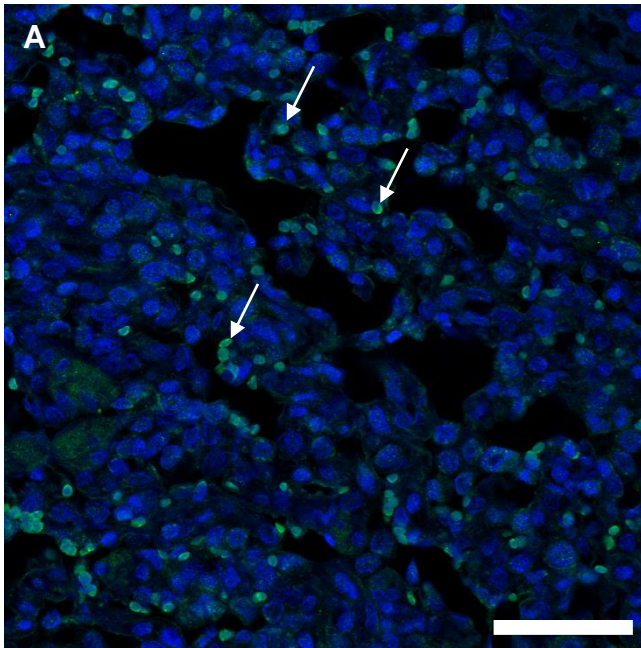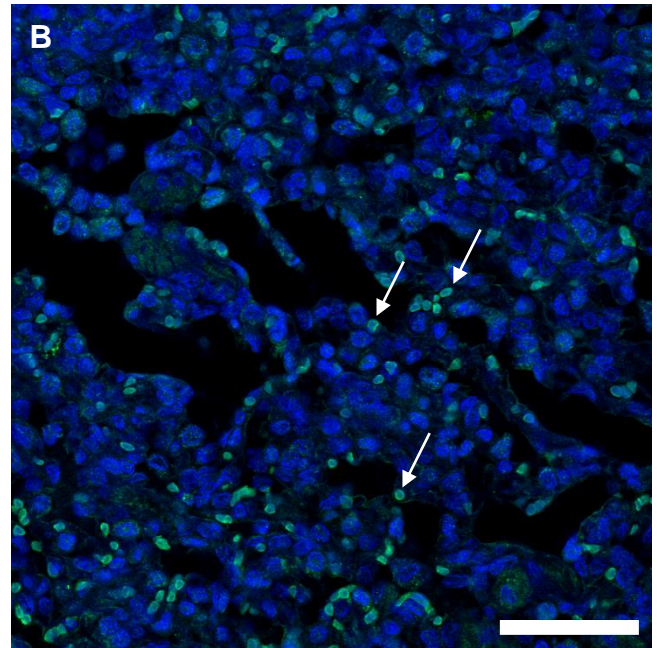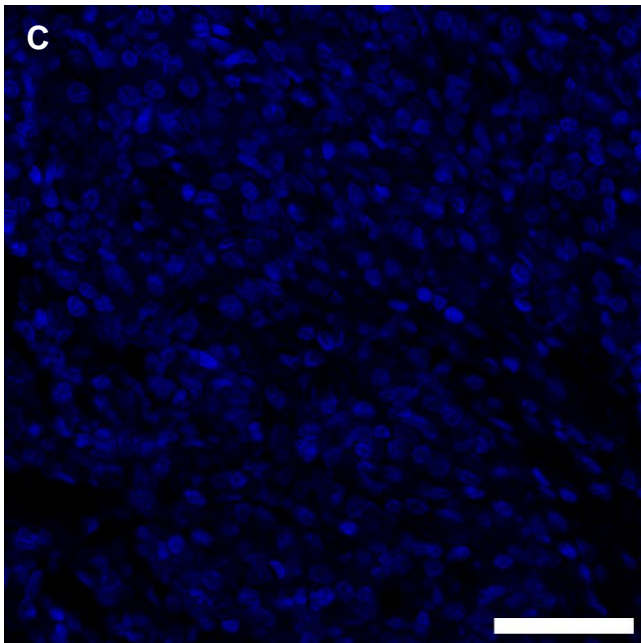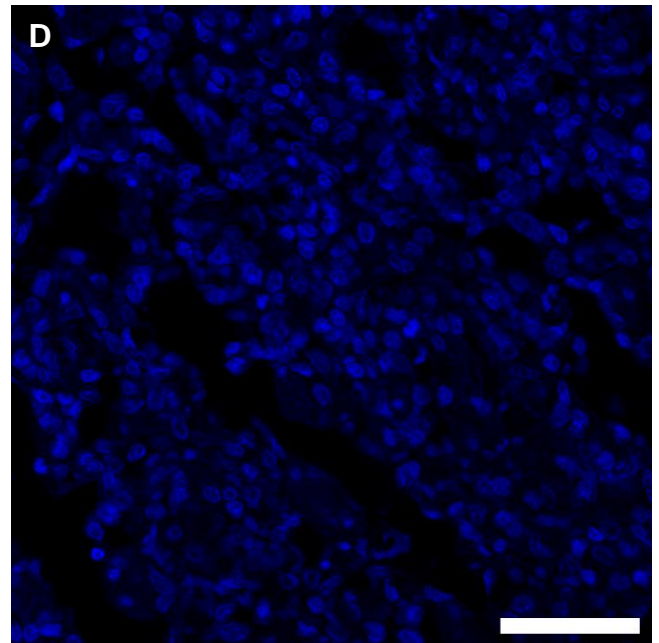

Supplement: Multimedia component 3 — Supplementary Figure 3. Immunolabeling of IL-4 receptor in inflamed lung regions. (A, C) Detection using DyLight™ 488-conjugated secondary antibody (green). (B, D) Detection using Alexa Fluor™ 488-conjugated secondary antibody (green). Nuclei were counterstained with DAPI (blue). Negative controls (absence of primary antibody) are shown in panels (C) and (D). White arrows indicate IL-4 receptor expression. Scale bar: 50 μm. [file mmc3.pdf]

# Autofluorescence of the blood red cells

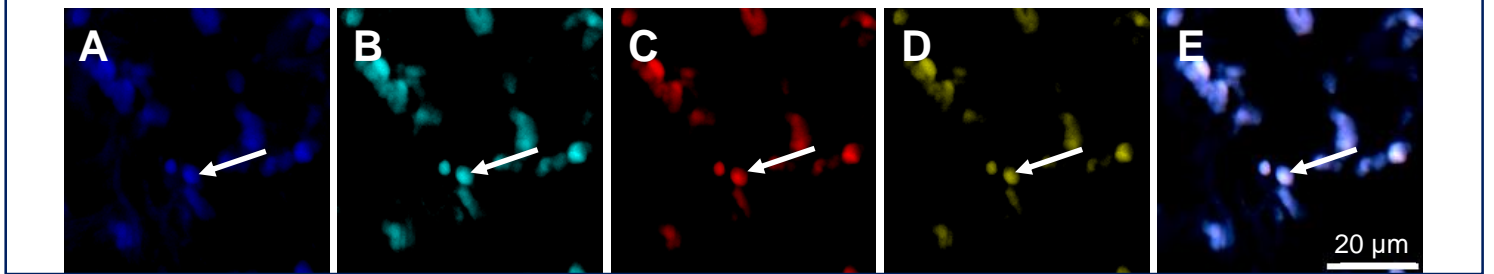

Supplement: Multimedia component 4 — Supplementary Figure 4. Visualization of intrinsic autofluorescence in the absence of antibody labeling. RBCs were identified based on morphology (small, round, anucleated cells within vascular lumens) and characteristic broad-spectrum autofluorescence. Signals were acquired following excitation at (A) 350 nm, (B) 488 nm, (C) 568 nm, and (D) 647 nm; (E) shows the merged image. [file mmc4.pdf]

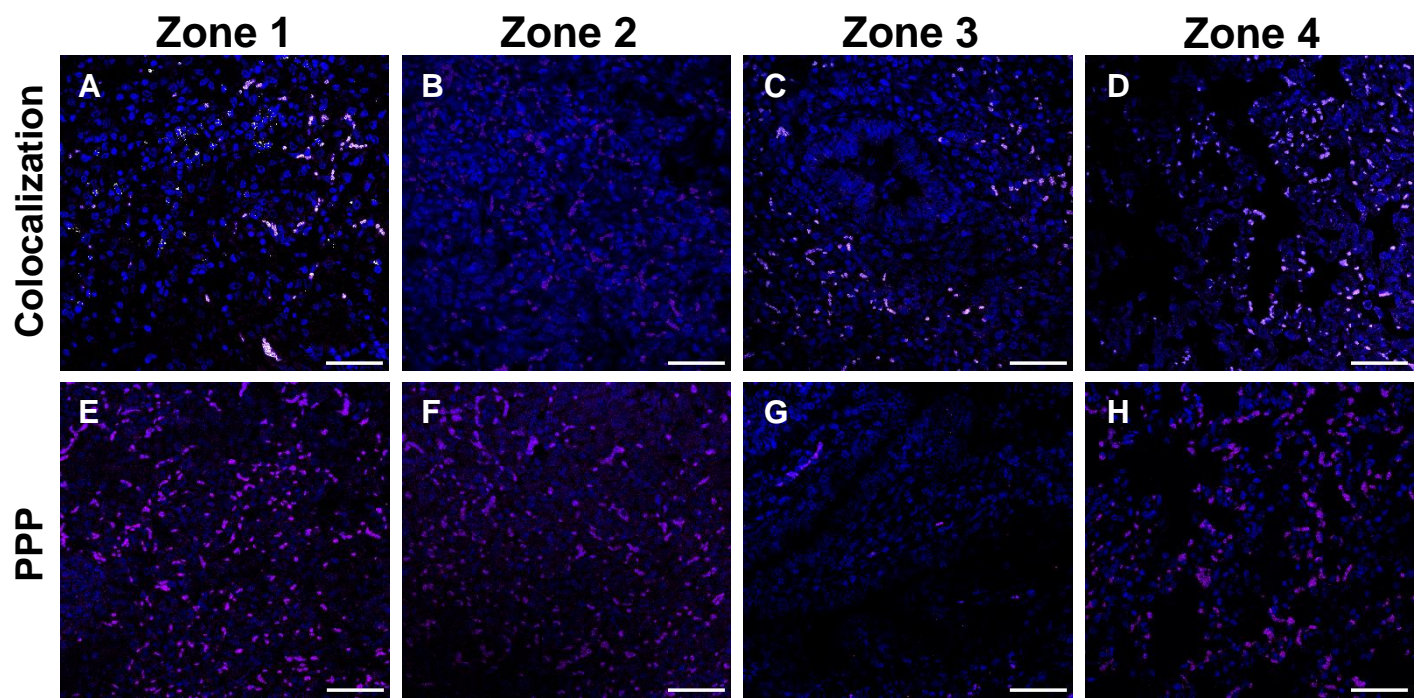

Supplement: Multimedia component 5 — Supplementary Figure 5. Negative controls for immunolabeling and PLA presented in Fig. 3. (A-D) Immunolabeling controls: (E-H) PLA controls (no primary antibodies). (A, E) Zone 1; (B, F) Zone 2; (C, G) Zone 3; (D, H) Zone 4. Signals in the absence of primary antibodies represent background and autofluorescence. IL-4R (Alexa Fluor 488, cyan), IL-4 (Alexa Fluor 568, red). Overlapping signals appear yellow. Autofluorescence (primarily erythrocytes) appears magenta. Nuclei (DAPI) appear blue. Scale bar: 50 μm. [file mmc5.pdf]

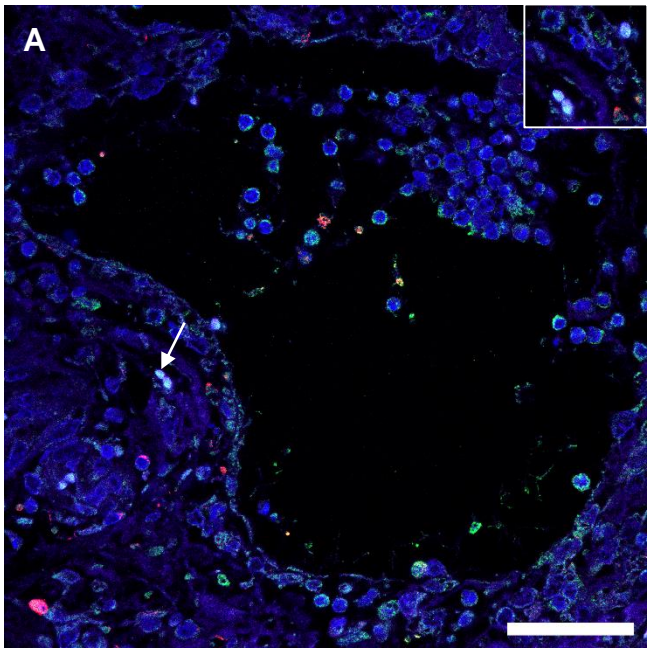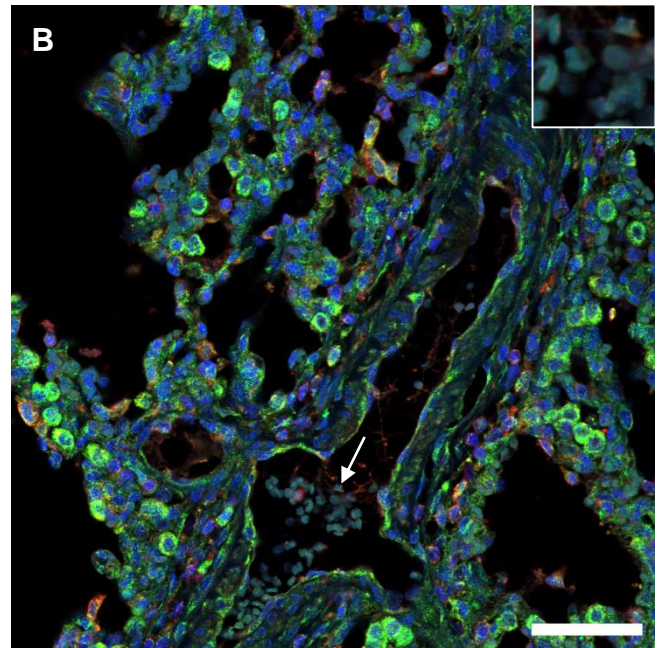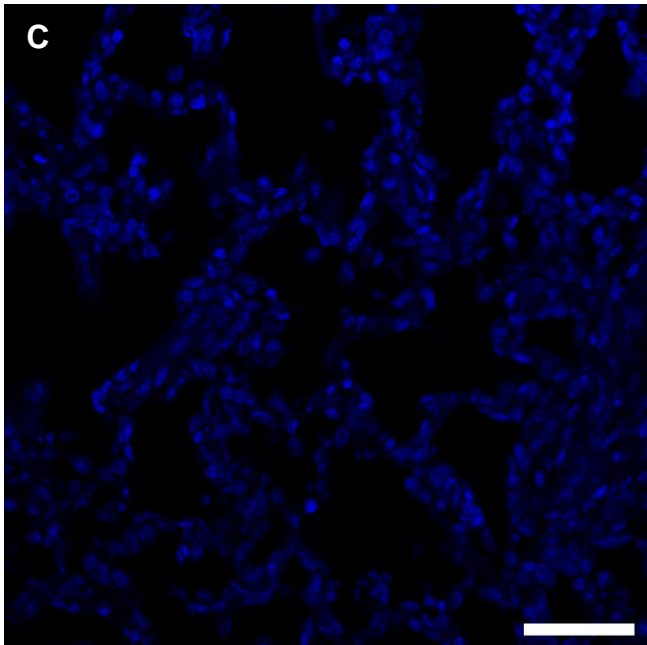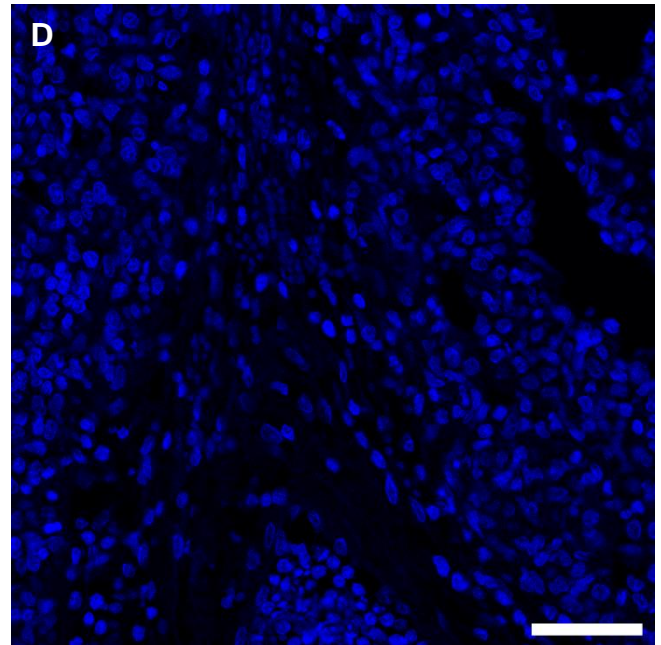

Supplement: Multimedia component 6 — Supplementary Figure 6. In situ co-localization of IL-4/IL-4R and TNF/TNFR1 in inflamed lung regions. (A, C) Immunolabeling of IL-4 and IL-4R in naturally infected pigs with lower respiratory tract disease. (B, D) Immunolabeling of TNF and TNFR1 in Zone 4 of pigs exposed to an organophosphorus agent. IL-4R and TNFR1 were detected using Alexa Fluor™ 488 (green), while IL-4 and TNF were detected using Alexa Fluor™ 568 (red). Nuclei were counterstained with DAPI (blue). The white square indicates a higher-magnification inset of the region highlighted by the arrow in the main image. Scale bar: 50 μm. [file mmc6.pdf]
